# Coordination Cages Selectively Transport Molecular Cargoes Across Liquid Membranes

**DOI:** 10.1021/jacs.1c04799

**Published:** 2021-08-02

**Authors:** Bao-Nguyen T. Nguyen, John D. Thoburn, Angela B. Grommet, Duncan J. Howe, Tanya K. Ronson, Hugh P. Ryan, Jeanne L. Bolliger, Jonathan R. Nitschke

**Affiliations:** † University of Cambridge, Department of Chemistry, Cambridge CB2 1EW, U.K.; ‡ Randolph-Macon College, Department of Chemistry, Ashland, Virginia 23005, United States

## Abstract

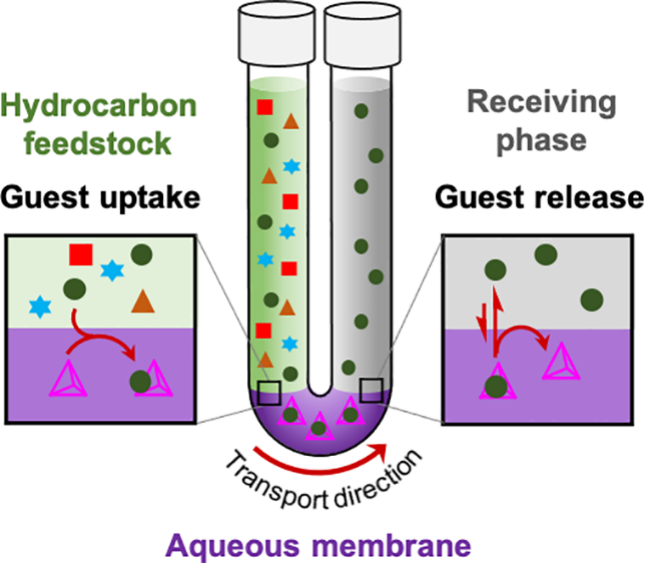

Chemical purifications are critical processes across many industries, requiring 10–15% of humanity’s global energy budget. Coordination cages are able to catch and release guest molecules based upon their size and shape, providing a new technological basis for achieving chemical separation. Here, we show that aqueous solutions of Fe^II^_4_L_6_ and Co^II^_4_L_4_ cages can be used as liquid membranes. Selective transport of complex hydrocarbons across these membranes enabled the separation of target compounds from mixtures under ambient conditions. The kinetics of cage-mediated cargo transport are governed by guest binding affinity. Using sequential transport across two consecutive membranes, target compounds were isolated from a mixture in a size-selective fashion. The selectivities of both cages thus enabled a two-stage separation process to isolate a single compound from a mixture of physicochemically similar molecules.

## Introduction

The binding properties of coordination cages^1−9^ in solution have been tailored to species ranging from gases^10−22^ to heavy metals,^23^ and neutral^24−28^ and charged^29−34^ compounds. A cage dissolved in one fluid phase is capable of extracting a guest from another immiscible one, without crossing the phase barrier.^35,36^ We envisaged that a cage dissolved in a water layer^37−39^ sandwiched between two organic solvent layers might be able to shuttle guests from one organic phase to the other. The aqueous cage layer would thus serve as a liquid membrane between the organic phases, with its guest-binding selectivity^40−42^ governing which molecules undergo transit.

Chemical separation using bulk liquid membranes^43−45^ has been seen as a promising prospect for many years. Such membranes consist of a fluid phase that is not miscible with two other liquids and separates them. They have been demonstrated to separate ions^45^ and heavy metals^46−49^ but not neutral molecules as yet. The use of such membranes for neutral-molecule separation may help enable the goal of purifying chemical mixtures at a lower energy cost than is currently possible.^50,51^ Membranes constructed using supramolecular principles have also shown a promising ability to selectively filter compounds based on size^52−54^ and charge^55^ differences.

**Figure 1:**
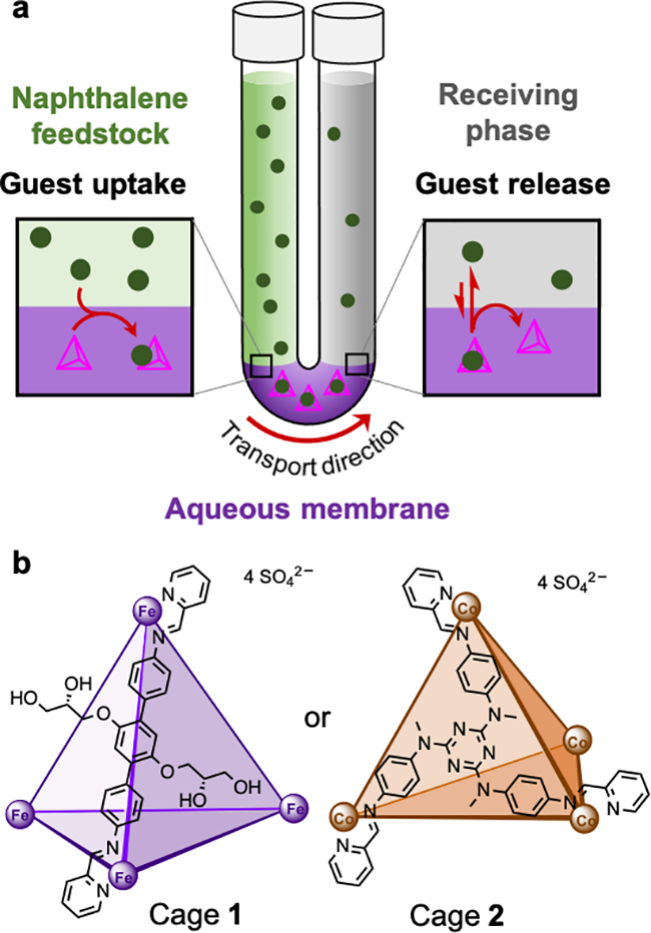
(a) The triphasic system configured in a U-shaped tube and our proposed mechanism of naphthalene transport by cages. Naphthalene (●) is encapsulated at the boundary between feedstock and aqueous membrane layers. The cages and their encapsulated cargoes diffuse through the aqueous layer to the receiving phase boundary. The encapsulated cargoes are then released from the cage cavities into the receiving organic phase. (b) Cages chosen for the aqueous membranes.

Here, we introduce the use of coordination cages as active carriers^56,57^ within liquid membranes. By selectively transporting neutral molecule guests across an aqueous layer, cages separate compounds from a mixture according to their binding affinity. As shown in Figure 1a, our system consists of a cage in an aqueous phase which acts as a membrane separating two organic layers, the feedstock and receiving phases. Cages within the liquid membrane selectively encapsulate target guest molecules, such as naphthalene, at the feedstock phase boundary, transport them across the membrane, and release them into the receiving phase. Guest uptake and release within this system thus occurs during thermodynamic equilibration between the free guests in the dodecane layers and the encapsulated ones in the cage layer. Guest transport from the stock arm to the receiving arm is driven by the concentration gradient between the two sides of the membrane, which favors guest transport from the higher concentration stock arm to the lower concentration receiving arm in a continuous process. This process could be used as the basis for a continuous chemical compound filtering system, in which coordination cages constantly encapsulate and release target compounds from stock mixtures to the receiving phases. Each host would continuously shuttle guest molecules, in contrast to a simpler biphasic batch extraction system where the extracted guest and host must first be separated before host reuse.

## Results and Discussion

### Naphthalene Transport by Cages **1** and **2** in Aqueous Bulk Membrane

The sulfate salts of tetrahedral Fe^II^_4_L_6_ cage **1**(58) and Co^II^_4_L_4_ cage **2**(41) (Figure 1b) were prepared as aqueous solutions and then loaded into the bottoms of U-shaped tubes (Figure 1a). In our initial studies, naphthalene was chosen as the target molecule due to its size-compatibility with cages **1** and **2**. Nevertheless, we anticipate that a wide range of other molecules, including other polycyclic aromatic hydrocarbons, could be selectively transported depending on the system. Solutions containing naphthalene dissolved in dodecane were then loaded into the feedstock arms, while pure dodecane was introduced into the receiving arms (Figure S11). Naphthalene was chosen as a guest molecule for these experiments because it was observed to bind to both cages **1** (Figure S27) and **2** (Figure S29) in water.^58^ Dodecane was chosen as the solvent because it readily dissolves naphthalene and has a boiling point of 216 °C, thus minimizing evaporative solvent loss.

**Figure 2:**
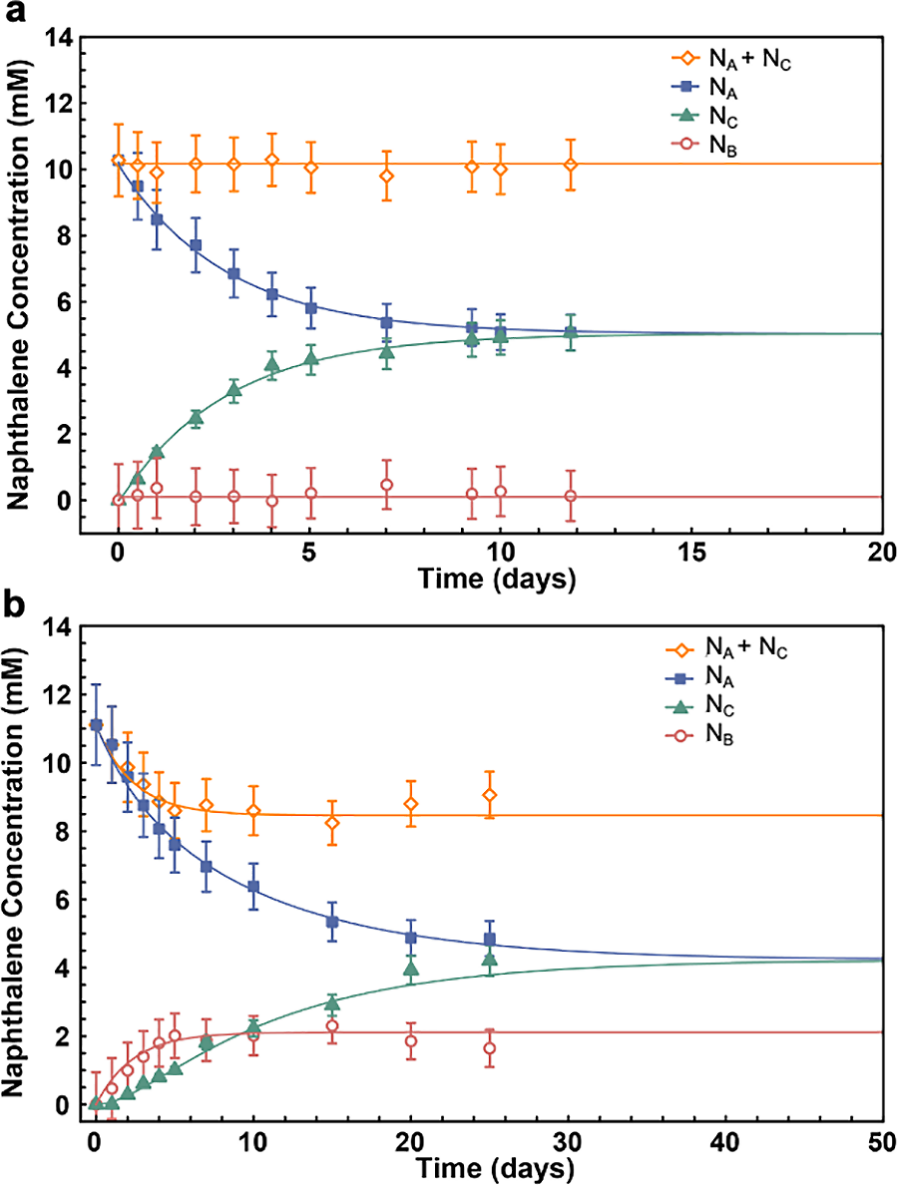
Transport of naphthalene from the feedstock to the receiving phase mediated (a) by cage **1** and (b) by cage **2**. Fitting to the transport model described in Supporting Information Section 5.2 provided molar flux values for guest transport through the aqueous membrane. (N_A_ = naphthalene concentration in the feedstock arm, N_B_ = in the cage layer, N_C_ = in the receiving arm).

Both cages **1** and **2** were observed to shuttle naphthalene across the aqueous membrane, with cage **1** (Figure 2a) acting more rapidly than cage **2** (Figure 2b). The transport data shown in Figure 2 were fitted to a three-state model (eq 1) in which the naphthalene is distributed between the feedstock arm (N_A_), the in-cage encapsulated state (N_B_) in the aqueous membrane, and the receiving arm (N_C_). The data for N_A_ and N_C_ were fit simultaneously using a nonlinear least-squares fit as implemented on *Mathematica* (Supporting Information, Section 5.2). The concentration of cage-encapsulated naphthalene, N_B_, was not measured but was rather inferred from mass balance based on the fitted intensities for N_A_ and N_C_.
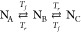
1

Fitting results (Figure 2) suggest that the time required for 50% of the naphthalene to transfer to the receiving arm is 2.0 days for cage **1** and 9.4 days for cage **2** (Table S3). We attribute the faster transport of naphthalene by cage **1** to more rapid guest ingress and egress from the cage framework. Assembled from edge-bridged ligands with flexible glycerol chains, cage **1** has more accessible apertures for guest ingress and egress than cage **2**. In contrast, the more enclosed framework of cage **2** presents a higher barrier to guest uptake and release.

Fitting to our model produced the rate constants given in Table S2, which are conveniently expressed in terms of molar fluxes *J*_*f*_ and *J*_*r*_ according to the equations *J*_*f*_ = *T*_*f*_ [*N*]·[*cage*] and *J*_*r*_ = *T*_*r*_ [*N*_B_], where *T*_*f*_ and *T*_*r*_ are the forward and reverse transport constants, and *N* is the naphthalene concentration in either the feedstock or receiving arm. For ingress into the aqueous cage **1** layer, *T*_*f*_^***1***^ = 0.157 ± 0.003 mM^–1^·day^–1^·cm^–2^, and for egress back into dodecane, *T*_*r*_^***1***^ = 12 ± 5 day^–1^·cm^–2^. Naphthalene transport through the aqueous membrane containing cage **2** was fitted to the same equations, resulting in molar flux transport constants *T*_*f*_^***2***^ = 0.045 ± 0.001 mM^–1^·day^–1^·cm^–2^ and *T*_*r*_^***2***^ = 0.14 ± 0.01 day^–1^·cm^–2^ for ingress and egress, respectively.

The sigmoidal rise in the concentration of naphthalene in the receiving arm when cage **2** serves as the carrier (Figure 2b) indicates an induction period, during which the host–guest intermediate builds up in the aqueous membrane, limiting the transfer rate. Such an induction period was observed in the case of the smaller *T*_*f*_ (0.045 mM^–1^·day^–1^·cm^–2^) for cage **2** but not in the case of the larger *T*_*f*_ (0.157 mM^–1^·day^–1^·cm^–2^) for cage **1**.

Our kinetic data showed that the molar flux for guest egress was greater than for guest ingress (*T*_*r*_ > *T*_*f*_), consistent with the observation that naphthalene release to the dodecane layers is more favorable than binding to the cages. The cage in the aqueous membrane must compete effectively with the dodecane solvent for naphthalene at the stock solution/aqueous phase boundary and still allow the release of naphthalene across the phase boundary into the receiving phase. Cage **1** was observed to be more effective than cage **2** at transporting naphthalene because it more readily took up (*T*_*f*_^***1***^ > *T*_*f*_^***2***^) and released guests (*T*_*r*_^***1***^ > *T*_*r*_^***2***^), reflecting the structural differences between the two cages discussed above.

To further investigate the role of the two cages in transporting naphthalene, a control experiment was conducted, where naphthalene was observed to diffuse across the liquid membrane at a much slower rate in the absence of a cage carrier (Figure S22) with *T*_*f*_ = 0.0049 ± 0.0004 mM^–1^·day^–1^·cm^–2^ and *T*_*r*_ = 0.14 ± 0.01 day^–1^·cm^–2^.

Although the transport coefficients for these processes are modest, the underlying physics allows the rate to be increased by simple modifications. For example, quadrupling the tube radius from *r* = 0.6 cm to 2.4 cm would increase the cross-sectional area and thus the flux by a factor of 16. This modification would reduce the naphthalene transport time without any change in the underlying functioning of the system. Additional rate enhancements resulted from increasing the amount of cage, as illustrated in Figure S24a.

### Selective Guest Filtration by Aqueous Membranes Containing Cages **1** and **2**

Having investigated the active transport of naphthalene across aqueous cage membranes, we began to explore systems wherein a series of cages selectively separated guests from a mixture. Because cages **1** and **2** have different internal volumes, we anticipated that they would transport different subsets of guest molecules. A feedstock containing naphthalene, mesitylene, *cis*-stilbene, and triisopropylbenzene (30 mM each) was thus chosen to demonstrate selectivity.

**Figure 3:**
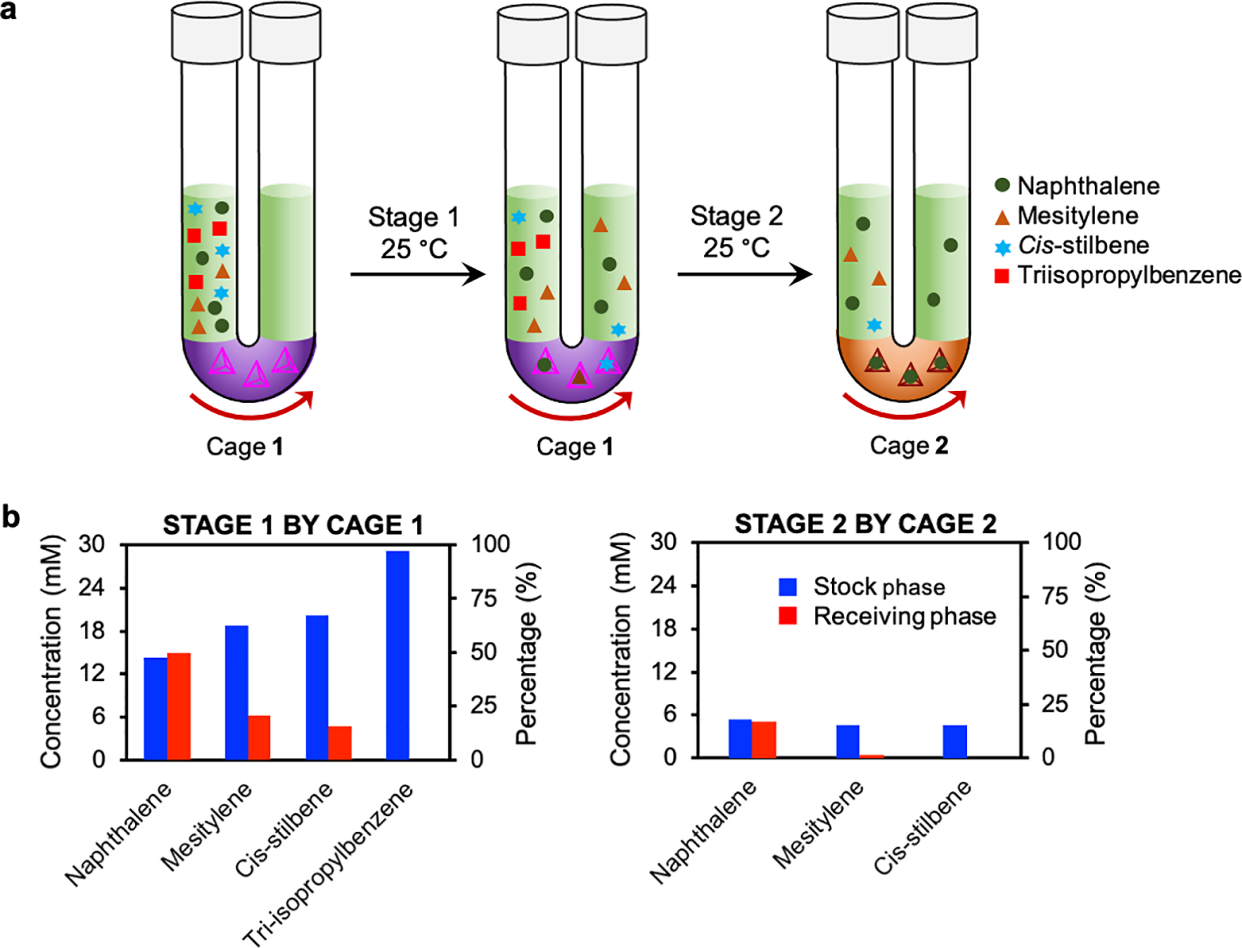
Illustration of the stepwise chemical separation. (a) A mixture of naphthalene, mesitylene, *cis*-stilbene and triisopropylbenzene was initially introduced to the feedstock arm of the first tube. Cage **1** selectively filtered naphthalene, mesitylene and *cis*-stilbene to the receiving arm, which then became the feedstock arm of the second stage, where cage **2** subsequently separated naphthalene from mesitylene and *cis-*stilbene. (b) Plots showing distribution of the compounds in the receiving phases following separation by cages **1** and **2**. Stage 1 took 43 days, and stage 2 took 25 days.

Our two-stage separation system is illustrated in Figure 3a. The first stage contained the larger cage **1** within the aqueous membrane, which allowed the transport of a set of larger guests. The second stage contained the smaller cage **2**, which bound smaller guests than cage **1**. The differences in binding selectivity between the two cages results primarily from their different sizes, with the central cavities of **1** and **2** estimated to be 418 (±2) Å^3^^58^ and 233 (±2) Å^3^,^41^ respectively. The filtration processes using the cage **1** and **2** membranes were set up in a stepwise manner. We envisaged that the larger cage **1** would selectively filter a larger number of guests from the stock layer while the smaller cage **2** would allow the passage of a smaller subset of guests following filtration by cage **1**.

In the first stage, after 43 days, approximately 50% of the naphthalene had been transported by cage **1**, resulting in 15 mM of the compound in both arms. Furthermore, mesitylene (6.3 mM, 21%) and *cis*-stilbene (4.7 mM, 16%) were transported into the receiving arm. No triisopropylbenzene was observed to undergo transport (Figure 3b).

Naphthalene was observed to transport most rapidly, and mesitylene was transported faster than *cis*-stilbene. We attribute the differences in guest transport rates to an interplay of kinetics and thermodynamics of guest binding. Naphthalene and mesitylene exited and entered **1** rapidly on the ^1^H NMR time scale, whereas the exchange of *cis*-stilbene and triisopropylbenzene was slow on the NMR timescale. Naphthalene and mesitylene thus kinetically outcompeted *cis*-stilbene to bind within **1**.

To probe the relative binding affinities of the guests for cage **1** in water, we carried out a guest-displacement assay. The following guests were added to cage **1** in water (1 mM, 0.5 mL): first triisopropylbenzene, next *cis*-stilbene, then mesitylene, and finally naphthalene. After the addition of each guest, the sample was analyzed by ^1^H NMR, to verify the progressive displacement of the encapsulated guests (Supporting Information Section 10.2, Figure S28).

The relative guest binding affinities thus help to account for the outcomes when multiple guests compete for transport. Naphthalene outcompeted *cis*-stilbene and mesitylene to bind within **1** and was thus transported preferentially. *Cis*-stilbene and mesitylene were transported next because they bound to cage **1** more strongly than triisopropylbenzene. Triisopropylbenzene bound only weakly to cage **1** and was not extracted from the stock layer by cage **1**. The transport of triisopropylbenzene was, therefore, not observed.

In stage 2 of the sequential guest purification system, the receiving phase from stage 1 containing naphthalene, mesitylene, and *cis*-stilbene was transferred into the feedstock arm of a new U-tube. An aqueous solution of cage **2** was added as the second membrane, and a new dodecane receiving layer was introduced (Figures 3a, S26). After 25 days, naphthalene had equilibrated across both arms, and mesitylene (0.3 mM, 1%) was also observed in the receiving arm, whereas no *cis*-stilbene was observed to transit.

Guests with stronger binding affinities impeded the transport of the weaker binding ones. To gauge competition between the guest compounds in the stock mixture, four control experiments were carried out, with either naphthalene, mesitylene, *cis*-stilbene, or triisopropylbenzene (30 mM each) present in the stock phase in the absence of the others. The experiments were analogous to the stage 1 separation using cage **1**. The guest transport to the receiving arms was monitored by ^1^H NMR for the first 7 days and after 43 days to match the duration of stage 1 separation.

In the absence of competing guests, a larger amount of mesitylene (7.1 mM, 24%) and *cis*-stilbene (8.2 mM, 27%) were independently transported to the receiving arms. Triisopropylbenzene was not transported even in the absence of the other guests. Notably, while more mesitylene than *cis*-stilbene was transported in stage 1, *cis*-stilbene showed faster independent transport in the control experiment, suggesting that the transport of *cis*-stilbene was accelerated in the absence of the other competing guests (Supporting Information, Figure S32). Our results highlight the effects of competition between the guests for the cage cavities and suggest that guests with stronger binding affinities impede the transport of those with weaker binding strengths.

## Conclusion

In summary, we demonstrated the use of soluble metal–organic cages as the active carriers in a new class of liquid membranes. Many more cages are available with varying shapes, sizes, and guest affinities, allowing our strategy to be broadly applied to many separation challenges. More robust cages used in the membrane layers will allow for a longer system lifespan and enable the use of elevated temperatures to speed up the guest transport. Many different solvents can also be used for these cages, including ionic liquids,^59^ potentially removing problems of membrane-phase evaporation and enabling membranes to be constructed that preclude transit outside of cage carriers. Similarly, a different combination of organic solvents used in the stock and receiving phases can also be further investigated to drive guest transport toward the receiving phase (see Supporting Information Section 6). Increasing the interface area and decreasing the transit distance will also increase the rates of mass flow through these membranes. The strategy outlined here can be developed into flow systems in which the immiscible organic and the aqueous membrane layers flow together to increase the interphase area, thus promoting effective host–guest interactions and increasing the rate of guest transit. A sequential filtration setup using multiple cage membranes will potentially allow for the separation of more than one target compound. Such systems may enable practical means of low-energy, high-fidelity chemical separation, as is required for the inevitable shift away from using hydrocarbons as fuels and toward using them as building blocks for new materials.

## Supporting Information Available

Synthesis and characterization data for the cages, experimental procedures for guest transport by the cage membranes, details of the kinetic modeling of guest transport, discussion of parameters which influence the rate of guest transport, host–guest studies of the cages (PDF)

## Accession Codes

CCDC 2047950 contains the supplementary crystallographic data for this paper. These data can be obtained free of charge via www.ccdc.cam.ac.uk/data_request/cif, or by emailing data_request@ccdc.cam.ac.uk, or by contacting The Cambridge Crystallographic Data Centre, 12 Union Road, Cambridge CB2 1EZ, UK; fax: +44 1223 336033.
