# Supplementary material for: Coordination Cages Selectively Transport Molecular Cargoes Across Liquid Membranes
Source: J Am Chem Soc. 2021 Aug 2;143(31):12175–80. doi: 10.1021/jacs.1c04799 (PMC8397303; doi:10.1021/jacs.1c04799)
Supplement: Supplementary file 1 — ja1c04799_si_001.pdf [file ja1c04799_si_001.pdf]

Supporting Information

Coordination Cages Selectively Transport Molecular Cargoes Across Liquid Membranes

*Bao-Nguyen T. Nguyen[1], John D. Thoburn[2], Angela B. Grommet[1], Duncan J. Howe[1], Tanya K. Ronson[1], Hugh P. Ryan[1], Jeanne L. Bolliger[1], Jonathan R. Nitschke[1]**

[1]University of Cambridge, Department of Chemistry, Cambridge, CB2 1EW, UK.

[2]Randolph-Macon College, Department of Chemistry, Ashland, VA, 23005, USA.

Table of Contents

1. Materials and Methods 2
2. Synthesis and characterization of cages **1** and **2** 3
   2.1. Assembly of Fe_4L_6, cage **1** 3
   2.2. Assembly of Co_4L_4 cage **2** 8
3. General setup procedure for experiments in U-shape tubes 10
4. Quantitative analysis by ^{1}H NMR 11
   4.1. Guest concentration and uncertainty calculation 12
   4.2. ^{1}H NMR experimental setup 16
5. Guest transport kinetic studies 20
   5.1. Kinetics experimental setup 20
   5.2. Modelling the transport mechanism 24
6. Outlook on potential solvent choices for receiving phase 28
7. Naphthalene transported by different concentrations of cage **2** 30
8. Naphthalene transported at varied feedstock concentration 31
9. Experimental setup for selective guest filtering experiments 32
10. Host-guest studies 36
   10.1. Naphthalene encapsulation by cage **1** in water 36
   10.2. Hierarchy of guest binding to cage **1** in water 37
   10.3. Guest binding to cage **2** in water 39
   10.4. Hierarchy of guest binding to cage **2** in water 41
11. Independent transport of the four guests by cage **1** 43
12. X-ray crystallography 44
13. References 46

1. Materials and Methods

Unless stated otherwise, all the reagents were purchased from commercial sources and used without further purification.

Nuclear Magnetic Resonance (NMR)

^{1}H NMR spectra were recorded using a 500 MHz AVIII HD Smart Probe and ^{19}F NMR were recorded using a Bruker 400 MHz Avance III HD Smart Probe NMR spectrometer. Chemical shifts for ^{1}H and ^{19}F are reported in ppm on the δ scale; ^{1}H and ^{19}F signals were referenced to the internal standards, coronene at 9.1 ppm and octafluoro-9,10-bis[4-(trifluoromethyl)phenyl]anthracene at −64 ppm, respectively. All the spectra were measured at 298 K, unless stated otherwise.

Electrospray Ionization Mass Spectrometry (ESI-MS)

ESI-MS was performed on a Micromass Quattro LC mass spectrometer (cone voltage 20 eV; desolvation temperature 348 K; ionisation temperature 338 K). High-resolution ESI mass spectra (HRMS) were obtained using Waters Synap system (capillary voltage 2 kV; cone voltage: 40-60V; desolvation temperature 293 K; source temperature 293 K).

2. Synthesis and characterization of cages 1 and 2

2.1. Assembly of Fe_4L_6 cage 1

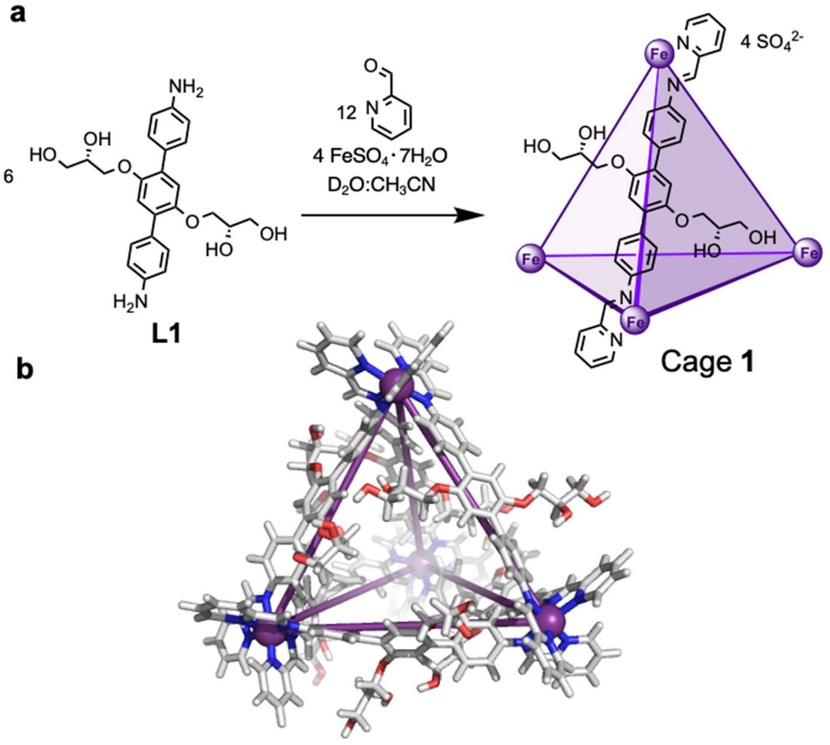

Figure S1: a, Synthetic scheme for cage **1**. **b**, Stick view of the molecular model of the cage using MM3 optimisation by SCIGRESS software.

The subcomponent (2*S*,2'*S*)-3,3'-((4,4"-diamino-[1,1':4',1"-terphenyl]-2',5'-diyl)bis(oxy))bis(propane-1,2-diol), **L1**, was synthesized following a reported procedure.[1] In a glovebox, to a screw capped vial containing **L1** (52.88 mg, 0.12 mmol, 6 equiv.), $FeSO_4 \cdot 7H_2O$ (22.24 mg, 0.08 mmol, 4 equiv.) and 2-pyridinecarboxaldehyde (22.83 μL, 0.24 mmol, 12 equiv.), a mixture of solvent D_2O:CH_3CN (10 mL, 1:1) was added. The reaction was stirred vigorously for 24 h at room temperature, forming a deep purple cage solution with a concentration of 2 mM. CH_3CN was subsequently removed by rotary evaporation and D_2O was added to the cage solution to make it up to a total volume of 10 mL. Excess 2-pyridinecarboxaldehyde was removed by washing the cage solution with ethyl acetate (2 x 10

mL). The remaining ethyl acetate in the aqueous cage solution was subsequently removed by rotary evaporation. Because the cage was observed to be unstable in the solid state, the cage was kept in D_2O solution and refrigerated for a longer storage time. ^{1}H NMR (400 MHz, D_2O, 298 K) δ_H (ppm) 8.93 (s, 12H, H_8), 8.46 (bm, 12H, H_{10}), 8.28 (bm, 12H, H_{12}), 7.62 (bm, 12H, H_{11}), 7.33 (bs, 12H, H_{13}), 7.21 (bm, 24H, H_5), 6.92 (bm, 12H, H_2), 5.52 (bm, 24H, H_6), 3.84 (bm, 24H, H_{14}), 3.66 (bm, 24H, H_{15}), 3.30 (bm, 24H, H_{16}). ^{13}C{^{1}H} NMR (126 MHz, D_2O, 298 K) δ_C (ppm) 175.3 (C_8), 159.2 (C_9), 156.5 (C_{13}), 150.6 (C_1), 150.2 (C_7), 140.7 (C_{12}), 139.2 (C_4), 131.8 (C_{10}), 131.3 (C_3), 131.0 (C_5), 130.3 (C_{11}), 122.2 (C_6), 117.2 (C_2), 71.0 (C_{15}), 70.5 (C_{14}), 63.6 (C_{16}). ESI-MS (positive) expected *m/z* = 491.9039 (+8), measured *m/z* = 491.9024 (+8).

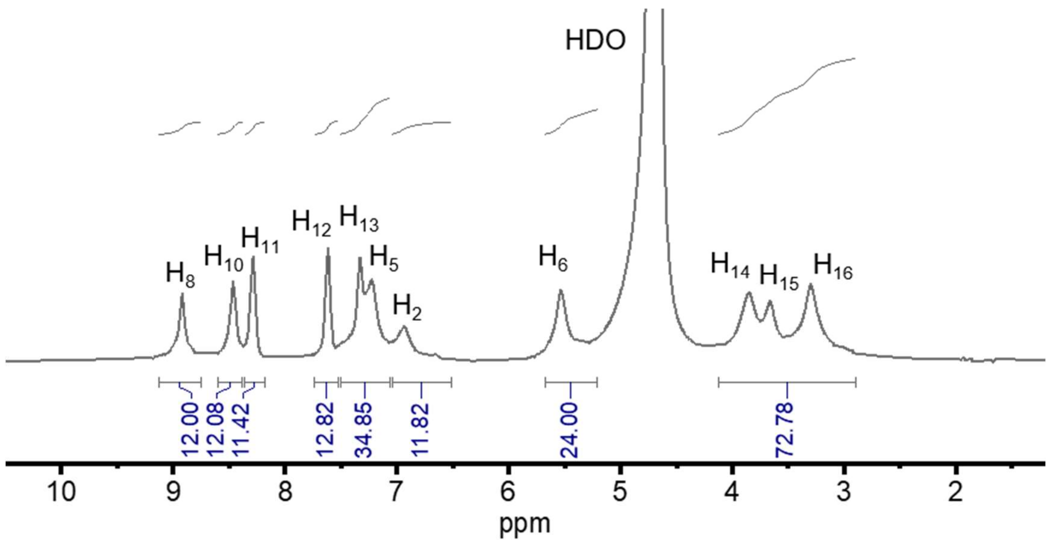

Figure S2: ^{1}H NMR spectrum (400 MHz, D_2O:CD_3CN (1:1), 298 K) of cage **1**.

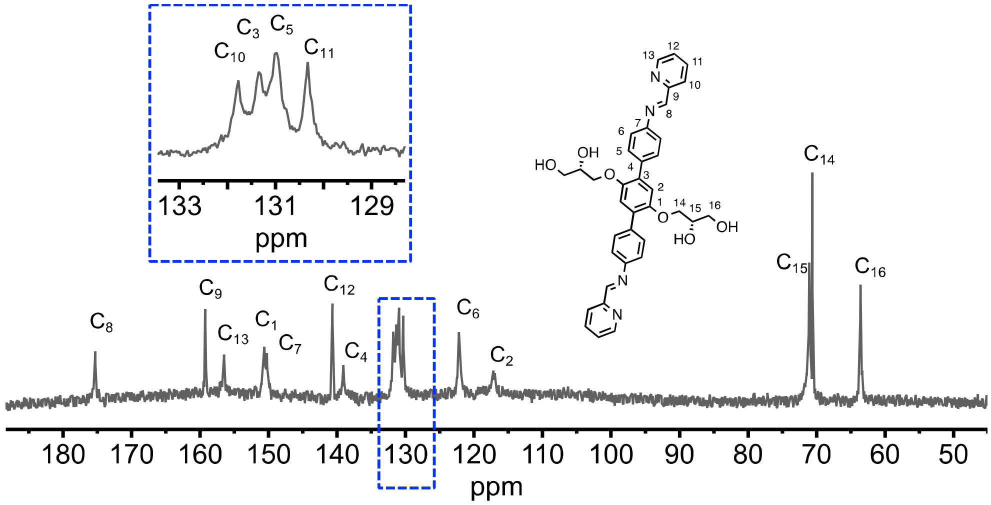

Figure S3: ^{13}C NMR spectrum (126 MHz, D_2O:CD_3CN (1:1), 298 K) of cage **1**.

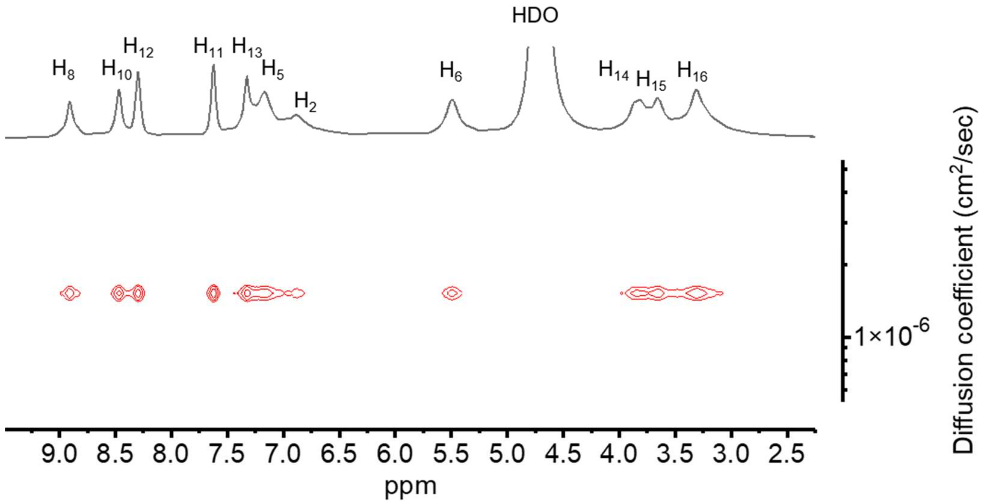

Figure S4: ^{1}H DOSY NMR (D_2O, 500 MHz, 298 K) of cage **1**.

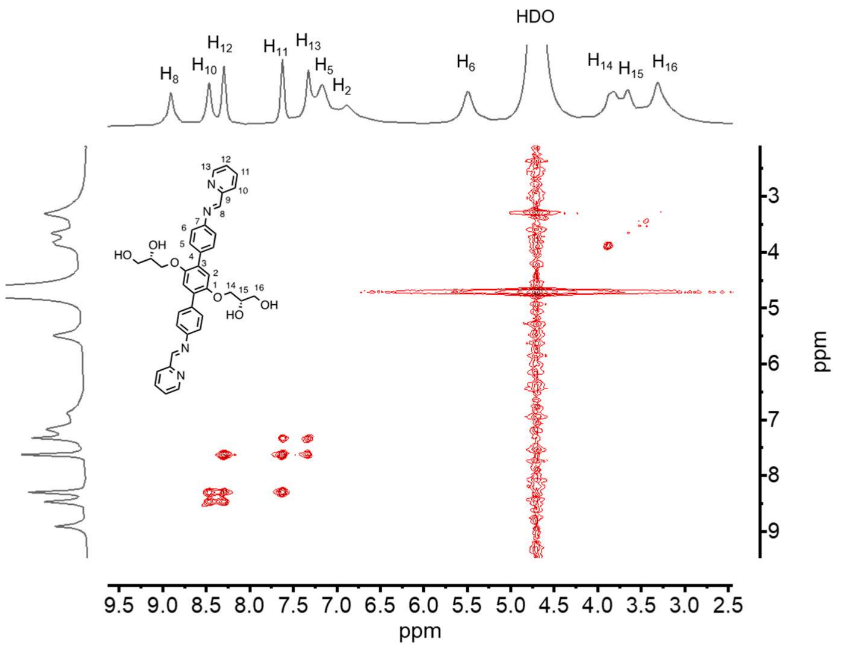

Figure S5: ^{1}H COSY NMR (D_2O, 500 MHz, 298 K) of cage **1**.

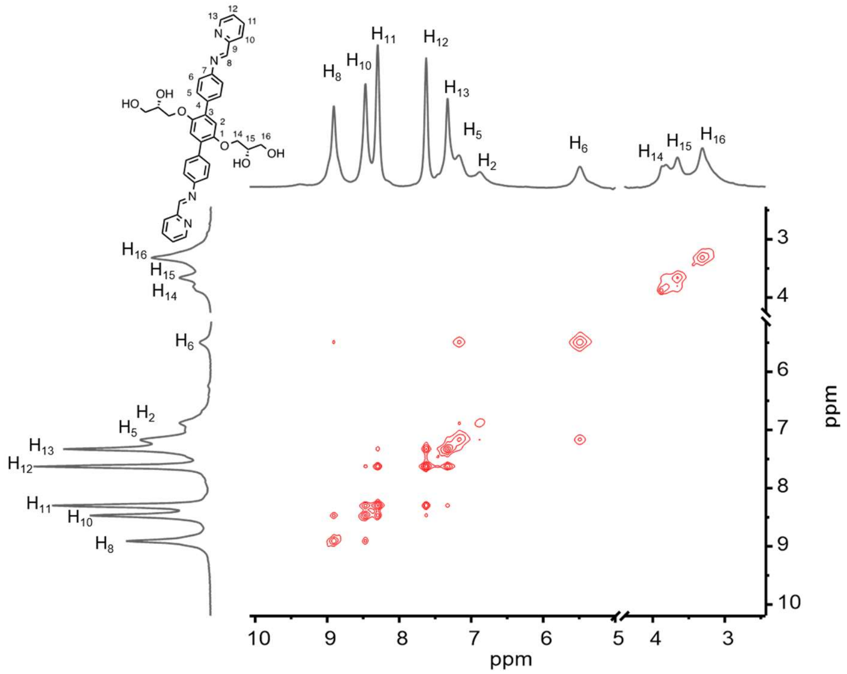

Figure S6: ^{1}H NOESY NMR (D_2O, 500 MHz, 298 K) of cage **1**.

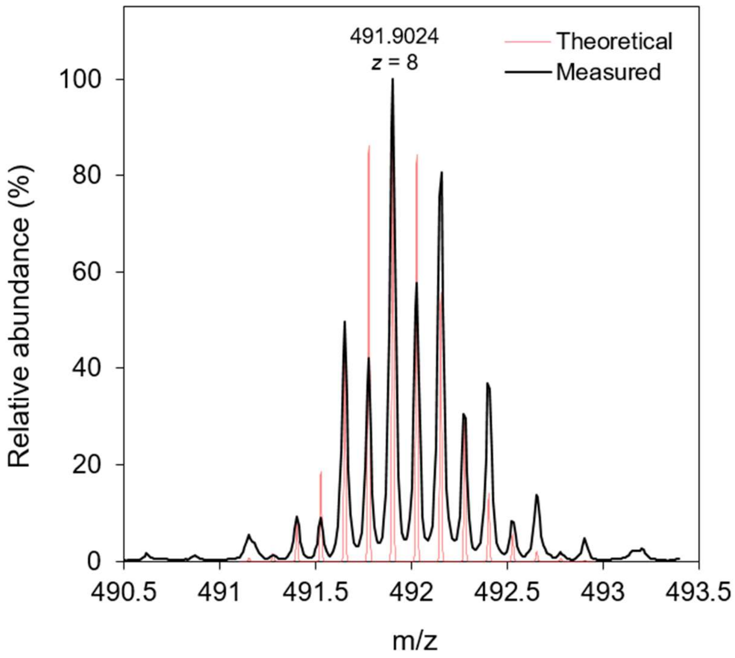

Figure S7: High-resolution ESI-mass spectrometry of **1**·[SO_4^{2-}]$_4$ showing z = +8 peak. ESI-MS (positive) expected m/z = 491.9039 (z = +8), measured 491.9024 (z = +8). The high resolution mass spectroscopy was obtained using sample with concentration of 0.005 mM in a mixture of D_2O:CH_3CN:MeOH (1:4:4).

The m/z = 491.9024 peak is assigned to the cage **1** $[Fe_4L_6]^{8+}$ complex. The deviation in the isotopic distribution is attributed to overlap of the $[Fe_4L_6]^{8+}$ ion and its homolytic fragment $[Fe_2L_3]^{4+}$, which is generated under the mass spectroscopy conditions. As harsh ionization conditions were used to obtain the high resolution ESI-MS, higher levels of fragmentation to form low charged +4 fragments was observed. No spectroscopic evidence suggests the formation of a $[Fe_2L_3]^{4+}$ complex in solution.

2.2. Assembly of Co_4L_4 cage 2

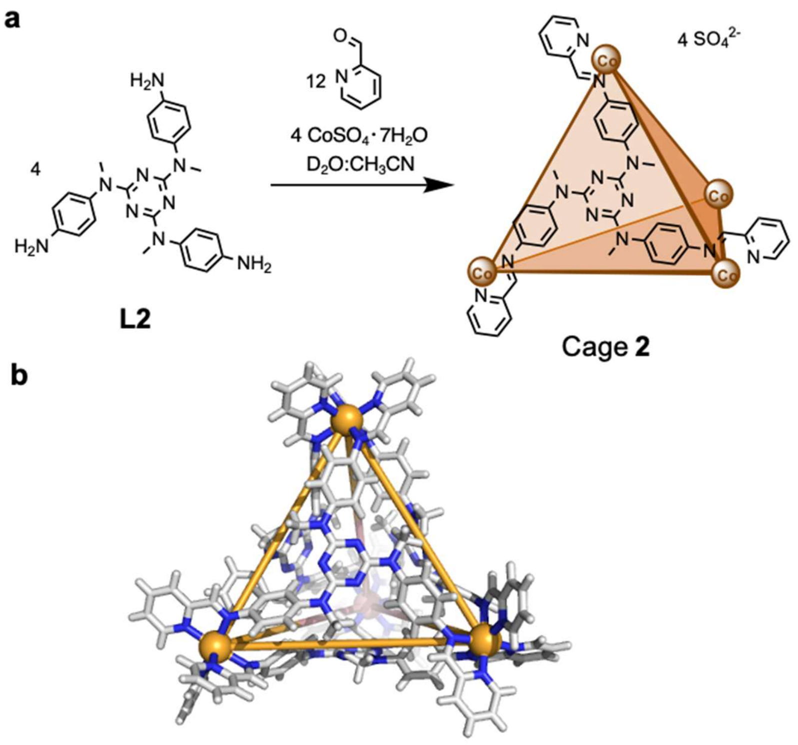

Figure S8: a, Synthetic scheme for cage **2**. **b**, Stick representation of the cage structure obtained from crystallographic data.

The subcomponent 1,3,5-triazine-2,4,6-triamine, *N,N',N''*-trimethyl-tris(4- aminophenyl), **L2**, was synthesized following a reported procedure.[2] A mixture of **L2** (141.3 mg, 0.32 mmol, 4 equiv.), $CoSO_4\cdot7H_2O$ (89.95 mg, 0.32 mmol, 4 equiv.) and 2-pyridinecarboxaldehyde (91.3 μL, 0.96 mmol, 12 equiv.) was dissolved in D_2O:CH_3CN (40 mL, 1:1). The reaction was stirred at room temperature for 24 hours, yielding a bright orange solution (2 mM). CH_3CN was subsequently evaporated, followed by the addition of D_2O (20 mL) to make the solution up to a total volume of 40 mL with 2 mM cage concentration. Excess 2-pyridinecarboxaldehyde can be removed by washing the cage solution with ethyl acetate (2 x 10 mL). The aqueous cage solution was put under rotary evaporation to remove the residual ethyl acetate. ^1H NMR (500 MHz, D_2O, 298 K) δ_H (ppm) 244.5 (H_7), 88.1 (H_9), 74.4 (H_{10}), 51.9 (H_{11}), 17.3 (H_{12}), 3.5 (H_4),

−5.2 (H_5), −21.2 (H_2). ESI-MS (positive) expected *m/z* = 815.2294 (+4), 527.4941 (+6), 383.6265 (+8), measured *m/z* = 811.9787, 526.4084, 385.1585.

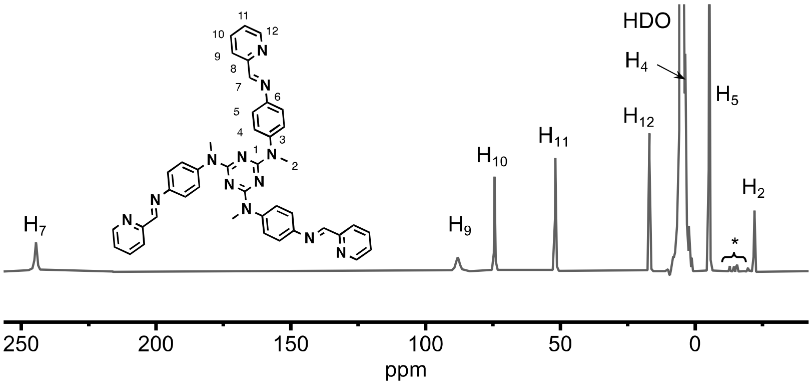

Figure S9: ^{1}H NMR spectrum (400 MHz, D_2O, 298 K) of cage **2**. (* = encapsulated 2-pyridinecarboxaldehyde peak signals).

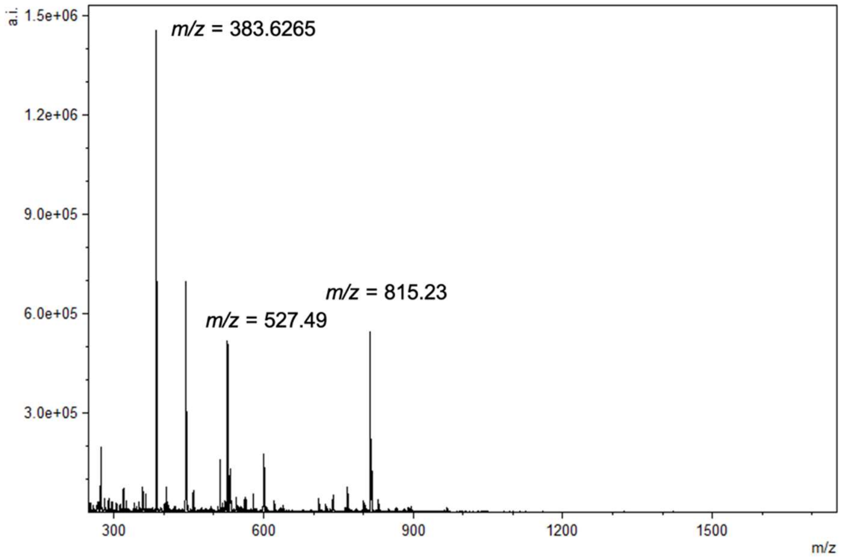

Figure S10: Low-resolution ESI-mass spectrum of **2**·$[SO_4^{2-}]_4$

3. General setup procedure for experiments in U-shape tubes

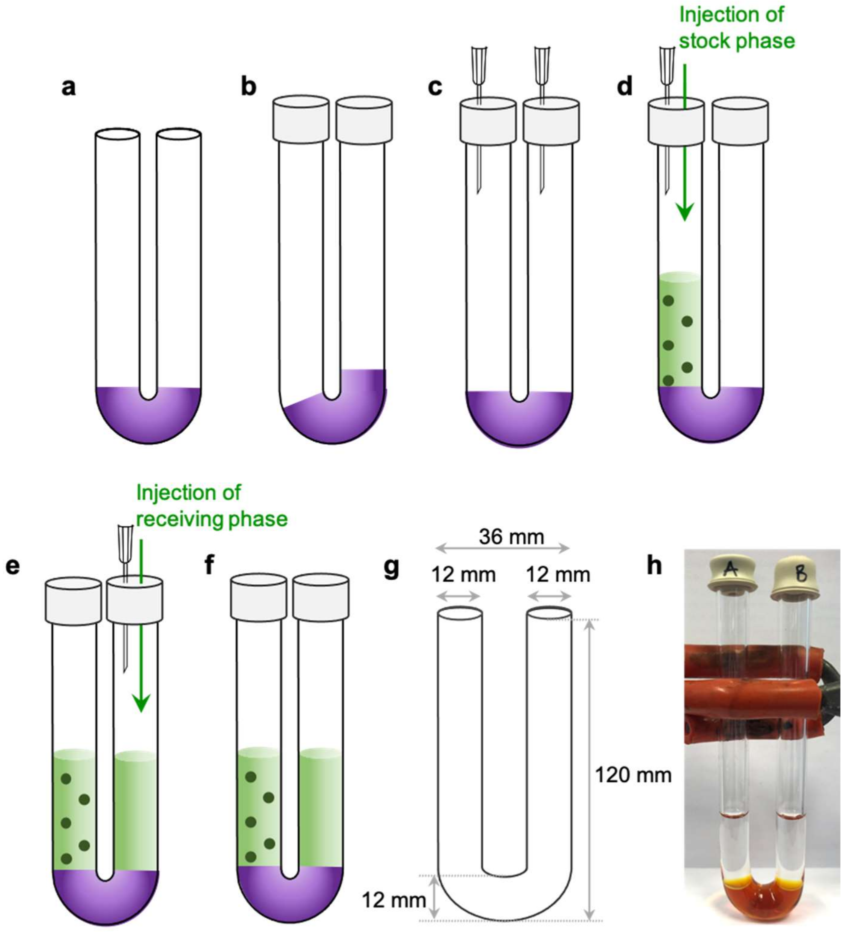

Figure S11: Experimental setup procedure in U-shaped tubes. **a**, A cage solution was introduced to the bottom of the tube. **b**, The tube was capped by the air-tight suba seals. **c**, Needles were inserted to release the pressure built up inside the tube and to make the solution levels even in the two tube arms. **d**, The needle in the receiving arm was removed to preserve a constant pressure in the arm when feedstock guest solution was injected to the other arm. **e**, The needle in the feedstock arm was subsequently removed to maintain a constant pressure within. A needle was re-inserted to the receiving arm, followed by the addition of the receiving phase. **f**, The needle was then removed. The experiment was stirred at room temperature at 250 rpm. **g**, The design and dimensions of the tubes. **h**, An example setup of an experiment with cage **2**.

The guest transport experiments were conducted in identical U-shaped tubes, which were designed as described in Figure S11. A constant volume of cage solution (2.5 mL) was first added to the bottom of the tube with a cylindrical magnetic stirrer bar (3 x 6 mm). The tube ends were subsequently closed by air-tight rubber suba seals, often causing the disturbance of

the cage layers. The cage solution levels in the two arms were equalised by inserting needles into the seals so that the pressure in both sides could equilibrate (Figure S11c). The needle in the receiving arm was removed to maintain a fixed air pressure inside when the feedstock solution was introduced to the other arm with the needle remaining inserted. The air replaced by the feedstock solution in the feedstock arm was released through the needle. The needle in the feedstock arm was subsequently removed, so that the pressure within could be preserved and the feedstock layer would not be disturbed when the receiving solution was introduced to the receiving arm. A new needle was inserted to the receiving arm, followed by the injection of a neat dodecane solution (Figure S11e). The needle was then removed. The air-tight suba seals will isolate the liquid phases inside the tube from the external environment and prevent solvent evaporation. The tube was placed on top of a magnetic stirring plate and was stirred at 250 rpm at room temperature.

4. Quantitative analysis by ^{1}H NMR

The concentration of compounds in the two arms was monitored by either ^{1}H NMR or ^{19}F NMR. To conduct NMR measurements, samples of 50 μL were taken out from the arms each time and were placed into micro NMR tubes (inner diameter = 2.4 ± 0.025 mm, outer diameter = 3.0 ± 0.025 mm, length = 100 ± 0.5 mm). Sealed capillary tubes (outer diameter = 1.8 – 2.0 mm, wall thickness = 0.28 – 0.32 mm, length = 100 mm) containing D_2O were inserted to the NMR tubes so that the experiments were locked with D_2O. After the measurement, the samples were re-injected back to the U-shaped tubes for the continuing experiments.

All the ^{1}H NMR spectra used for quantitative studies were referenced to coronene as the internal standard, at 9.1 ppm. The coronene integral was measured from f1 = 9.15 ± 0.005 ppm to f1 = 9.05 ± 0.005 ppm and was normalized to 100, corresponding to twelve protons.

4.1. Guest concentration and uncertainty calculation

Naphthalene uncertainty calculation

Naphthalene peaks were integrated from f1 = 8.06 ± 0.005 ppm to f1 = 7.95 ± 0.005 ppm, corresponding to $4H_a$, and f1 = 7.68 ± 0.005 ppm to f1 = 7.58 ± 0.005 ppm, corresponding to $4H_b$. The concentration of naphthalene presence in the organic phases were calculated by Formula 1, referencing to coronene (0.25 mM).

$$[\text{Naphthalene}] = \frac{\text{N} \times 0.25\ \text{mM} \times 12}{\int \text{coronene} \times 4} \quad (1)$$

N is the average integral of H_a and H_b, corresponding to 4 protons.

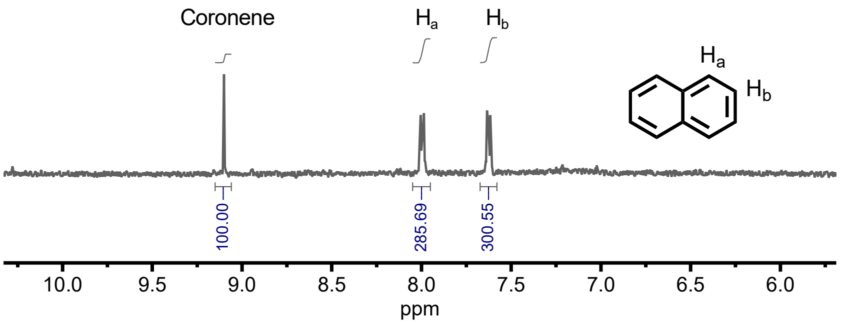

Figure S12: ^{1}H NMR spectrum (500 MHz, D_2O, 298 K) of a sample from the receiving arm containing naphthalene and coronene. The integrals of the naphthalene peak signals were determined relative to coronene at 9.1 ppm, which was given a normalised integral of 100.

The uncertainties of the data points were calculated based on error propagation (Formula 2), induced from the error of ^{1}H NMR integration, which is ± 10 %.[3,4]

$$\partial[\text{Naphthalene}] = \partial\left(\frac{\text{N} \times 0.25\ \text{mM} \times 12}{\int \text{coronene} \times 4}\right) = \frac{0.25 \times 12}{4} \times \partial\left(\frac{\text{N}}{\int \text{coronene}}\right)$$

in which:

$$\partial\left(\frac{\text{N}}{\int \text{coronene}}\right) = \frac{\text{N}}{\int \text{coronene}} \times \sqrt{\left(\frac{\partial \text{N}}{\text{N}}\right)^2 + \left(\frac{\partial \int \text{coronene}}{\int \text{coronene}}\right)^2}$$

$$\frac{\partial \int \text{coronene}}{\int \text{coronene}} = 0.1\ ;$$

$$\partial\mathrm{N} = \frac{1}{2} \times \sqrt{(\partial\mathrm{H_a})^2 + (\partial\mathrm{H_b})^2} = \frac{1}{2} \times \sqrt{(0.1 \times \mathrm{H_a})^2 + (0.1 \times \mathrm{H_b})^2}.$$

$$\partial[\text{Naphthalene}] = \frac{0.25 \times 12}{4} \times \frac{\mathrm{N}}{\int\text{coronene}} \times \sqrt{\left(\frac{\partial\mathrm{N}}{\mathrm{N}}\right)^2 + \left(\frac{\partial\int\text{coronene}}{\int\text{coronene}}\right)^2}$$

$$\partial[\text{Naphthalene}] = \frac{3}{4} \times \frac{\mathrm{N}}{100} \times \sqrt{\left(\frac{\partial\mathrm{N}}{\mathrm{N}}\right)^2 + (0.1)^2} \qquad (2)$$

Cis-stilbene uncertainty calculation

Similarly, *cis*-stilbene signals were measured from f1 = 7.49 ± 0.005 ppm to 7.29 ± 0.005 ppm, corresponding to 10H$_c$, and from f1 = 6.84 ± 0.005 ppm to 6.75 ± 0.005 ppm, corresponding to 2H$_d$. The concentration values of *cis*-stilbene were calculated using Formula 3.

$$[\textit{cis}\text{-stilbene}] = \frac{\mathrm{S} \times 0.25\ \mathrm{mM} \times 12}{\int\text{coronene}}\ ;\ \text{in which}\ \ \mathrm{S} = \frac{\int\mathrm{H_c} + \int\mathrm{H_d}}{12} \qquad (3)$$

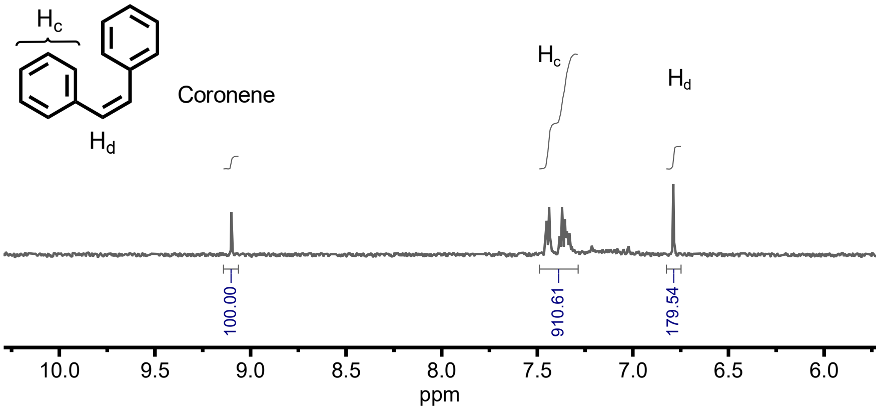

Figure S13: ^{1}H NMR spectrum (500 MHz, D_2O, 298 K) of a sample from the receiving arm containing *cis*-stilbene and coronene. The integrals of the *cis*-stilbene peak signals were determined relative to coronene at 9.1 ppm with a normalised integral of 100.

The error propagation of *cis*-stilbene concentration was calculated using Formula 4.

$$\partial[\textit{cis}\text{-stilbene}] = \partial\left(\frac{\mathrm{S} \times 0.25\ \mathrm{mM} \times 12}{\int\text{coronene}}\right) = 3 \times \partial\left(\frac{\mathrm{S}}{\int\text{coronene}}\right)$$

in which:

$$\partial\left(\frac{\mathrm{S}}{\int \mathrm{coronene}}\right) = \frac{\mathrm{S}}{\int \mathrm{coronene}} \times \sqrt{\left(\frac{\partial \mathrm{S}}{\mathrm{S}}\right)^2 + \left(\frac{\partial \int \mathrm{coronene}}{\int \mathrm{coronene}}\right)^2}$$

$$\frac{\partial \int \mathrm{coronene}}{\int \mathrm{coronene}} = 0.1\,;$$

$$\partial \mathrm{S} = \frac{1}{12} \times \sqrt{(\partial \mathrm{H}_c)^2 + (\partial \mathrm{H}_d)^2} = \frac{1}{12} \times \sqrt{(0.1\ \times \mathrm{H}_c)^2 + (0.1\ \times\ \mathrm{H}_d)^2}.$$

$$\partial[cis\text{-stilbene}] = 3 \times \frac{\mathrm{S}}{\int \mathrm{coronene}} \times \sqrt{\left(\frac{\partial \mathrm{S}}{\mathrm{S}}\right)^2 + \left(\frac{\partial \int \mathrm{coronene}}{\int \mathrm{coronene}}\right)^2}$$

$$\partial[cis\text{-stilbene}] = 3 \times \frac{\mathrm{S}}{100} \times \sqrt{\left(\frac{\partial \mathrm{S}}{\mathrm{S}}\right)^2 + (0.1)^2} \qquad (4)$$

Mesitylene uncertainty calculation

Mesitylene concentration was calculated from its integral values using Formula 5. Integration of the mesitylene peak was measured from f1 = 7.00 ± 0.005 ppm to 6.92 ± 0.005 ppm, corresponding to 3H_e. M is the H_e integral value.

$$[\mathrm{Mesitylene}] = \frac{\mathrm{M} \times 0.25\ \mathrm{mM} \times 12}{3 \times \int \mathrm{coronene}} \qquad (5)$$

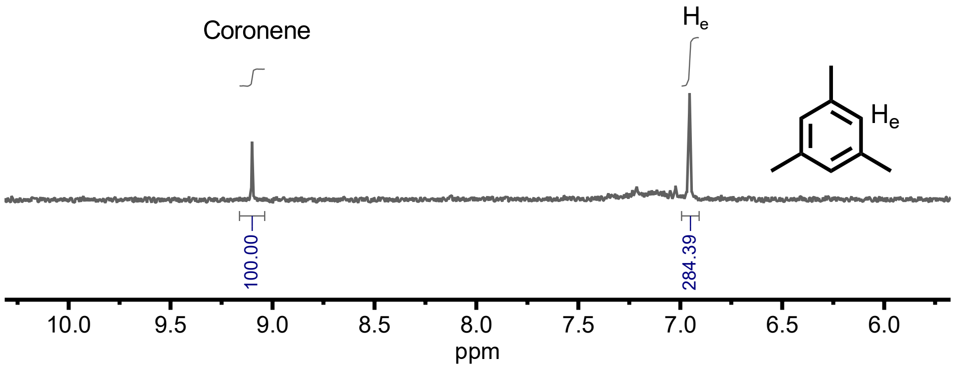

Figure S14: ^{1}H NMR spectrum (500 MHz, D_2O, 298 K) of a sample from the receiving arm containing mesitylene and coronene. The integration of mesitylene peak signals was determined relative to coronene at 9.1 ppm with a normalised integration of 100.

The uncertainty of mesitylene concentration was calculated using Formula 6.

$$\partial[\text{Mesitylene}] = \partial\left(\frac{\text{M} \times 0.25\ \text{mM} \times 12}{3 \times \int\text{coronene}}\right) = \frac{0.25\ \times\ 12}{3} \times \partial\left(\frac{\text{M}}{\int\text{coronene}}\right)$$

in which:

$$\partial\left(\frac{\text{M}}{\int\text{coronene}}\right) = \frac{\text{M}}{\int\text{coronene}} \times \sqrt{\left(\frac{\partial\text{M}}{\text{M}}\right)^2 + \left(\frac{\partial\int\text{coronene}}{\int\text{coronene}}\right)^2}$$

$$\frac{\partial\int\text{coronene}}{\int\text{coronene}} = 0.1\ ;\quad \frac{\partial\text{M}}{\text{M}} = 0.1\ ;$$

$$\partial[\text{Mesitylene}] = \partial\left(\frac{\text{M}}{\int\text{coronene}}\right)$$

$$\partial[\text{Mesitylene}] = \frac{\text{M}}{\int\text{coronene}} \times \sqrt{(0.1)^2 + (0.1)^2} \tag{6}$$

Triisopropylbenzene uncertainty calculation

Triisopropylbenzene concentration was calculated from its integral values using Formula 7. The compound integration measured from f1 = 7.15 ± 0.005 ppm to 7.05 ± 0.005 ppm, corresponding to $3H_f$. T is H_f integral.

$$[\text{Triisopropylbenzene}] = \frac{\text{T} \times 0.25\ \text{mM} \times 12}{3\ \times \int\text{coronene}} \tag{7}$$

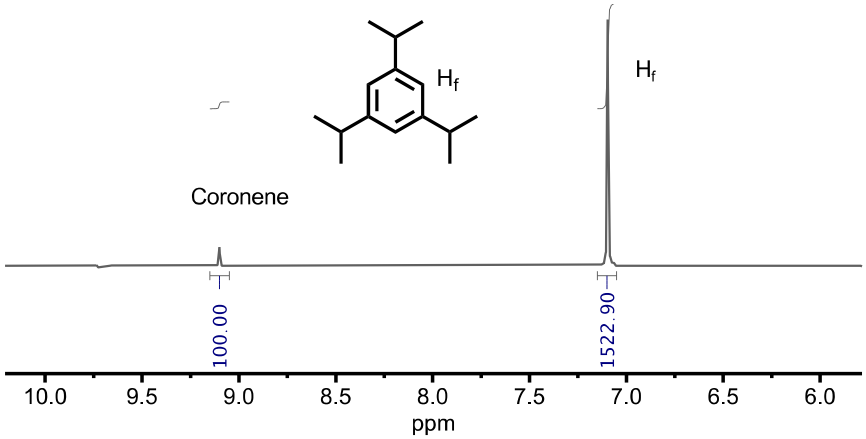

Figure S15: ^{1}H NMR spectrum (500 MHz, D_2O, 298 K) of a sample from the receiving arm containing triisopropylbenzene and coronene. The integration of mesitylene peak signals were determined relative to coronene at 9.1 ppm with a normalised integral of 100.

The uncertainty of triisopropylbenzene concentration was calculated using Formula 8.

$$\partial[\text{Triisopropylbenzene}] = \partial\left(\frac{\text{T} \times 0.25\ \text{mM} \times 12}{3 \times \int\text{coronene}}\right) = \frac{0.25 \times 12}{3} \times \partial\left(\frac{\text{T}}{\int\text{coronene}}\right)$$

in which:

$$\partial\left(\frac{\text{T}}{\int\text{coronene}}\right) = \frac{\text{T}}{\int\text{coronene}} \times \sqrt{\left(\frac{\partial\text{T}}{\text{M}}\right)^2 + \left(\frac{\partial\int\text{coronene}}{\int\text{coronene}}\right)^2}$$

$$\frac{\partial\int\text{coronene}}{\int\text{coronene}} = 0.1\ ; \quad \frac{\partial\text{T}}{\text{T}} = 0.1\ ;$$

$$\partial[\text{Triisopropylbenzene}] = \partial\left(\frac{\text{T}}{\int\text{coronene}}\right)$$

$$\partial[\text{Triisopropylbenzene}] = \frac{\text{T}}{\int\text{coronene}} \times \sqrt{(0.1)^2 + (0.1)^2} \qquad (8)$$

4.2. ^{1}H NMR experimental setup

Most of the NMR samples contain a large amount of dodecane solvent. A typical ^{1}H NMR experiment recorded over a 0 – 12 ppm range resulted in the high-intensity dodecane peaks distorting the relatively small guest peak signals. To avoid the influence of the dodecane, solvent suppression methods were initially attempted, as used successfully in previous studies.[5,6] However in this work, the solvent suppression methods distorted the baseline and gave rise to artefact peaks which prevented accurate analysis of the signals of interest.

We found that by simply changing the irradiation frequency (O1P value = 15 ppm) so that the dodecane signals do not lie within the observation 'window' the spectrometers digital filtration is sufficiently good to produce a result largely free from any distortion. Any lingering baseline deviations can be removed by only transforming a subset of the full frequency range observed and then applying a baseline correction ablative function. We found the signal to noise ratio sufficiently good to get accurate results in a reasonable time. The experiments were performed on a Bruker 500MHz AVIII HD spectrometer, equipped with a broadband (31P-109Ag) 'BBFO' probe, running Topspin 3.2.

A nominal 30° pulse was used, using the software default 'zg30' pulse program. An irradiation frequency corresponding to 15 ppm was used, digitising a 10,000 Hz frequency range with a 64 K resolution over 3.28 s. A relaxation delay of 1 s was used, to give a total pulse recycle time of 4.28 s. Every NMR experiment was recorded with a constant 32 scans, using a constant receiver gain of 12.7. The results were processed using the MNova (versions 14.1.1-24571 and 11.0.4-18998) software package and were Fourier transformed at the observed resolution of 64 K, using an exponential 'line broadening' function of 1 Hz. Manual phase correction was used, along with an 'ablative' baseline correction algorithm to allow accurate integration with the software's auto-linear function. The accuracy of the baseline correction and therefore integration, can further be improved by only transforming a limited set of frequencies from 9.8 to 6.6 ppm.

To evaluate the accuracy of the NMR experiments in quantifying concentrations, a test experiment was conducted. Five solutions containing known concentrations of naphthalene (23, 11.5, 5.75, 2.875 and 1.15 mM) in dodecane were prepared by volumetric dilution from a feedstock. The test solutions contain coronene (0.25 mM) as internal standard. ^{1}H NMR spectra of five samples of each solution were measured using the experimental parameters described above, using 3 mm NMR tubes containing a D_2O capillary.

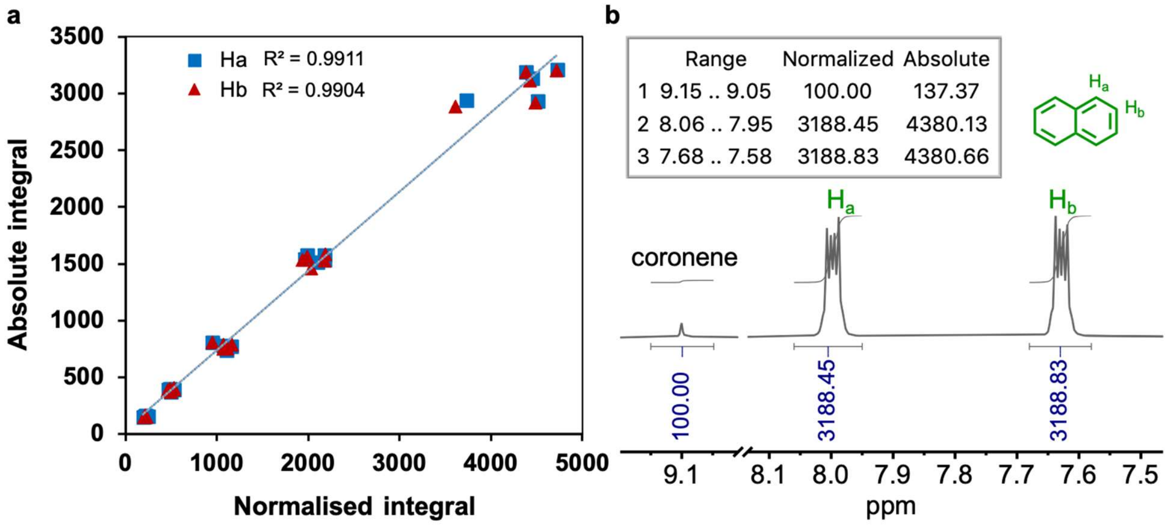

Figure S16: a, Graph of the absolute integral vs. normalised integral for the naphthalene proton signals. The linear fit suggests that the absolute and the normalised values are linearly proportional. **b**, Example of an ^{1}H NMR spectrum with the peak normalised and absolute integrals reported.

It is noteworthy that the relative accuracy of comparing integrals depends on the difference between T1 values. As we used 30° pulses, rather than 90° pulses, we expect the spins to relax sufficiently. In addition, having a D_2O capillary may affect the intensity of the signal observed, depending on its position within the tube and its width. In these experiments, D_2O capillaries were closely fitted into the NMR tubes and were therefore always keep in a vertical position.

Both relative and absolute integral values of naphthalene will also depend on the accuracy of the feedstock/test solution preparation. To evaluate the accuracy of the solution preparation and data processing, a graph of the absolute versus the normalised integrals of naphthalene proton signals, H_a and H_b, were plotted and linearly fitted (Figure S16b). The R^2 values are close to 1, suggesting that the normalised and the absolute integrals are linearly proportional.

The concentration of naphthalene was re-calculated referenced to coronene (0.25 mM). The deviation of the calculated concentration values from the prepared one was identified and presented as a percentage. It was observed that the ^{1}H NMR experimental setup allowed the quantitative study of naphthalene concentration within an accuracy of 100 ± 5% (Table S1).

Table S1: Studies of concentration deviation of naphthalene measured by NMR peak integration in reference to coronene (0.25 mM), compared to the prepared concentration of the samples.

Prepared concentration / mM	Sample numbers	$\int H_a$	$\int H_b$	$\frac{\int H_a + \int H_b}{2}$	Measured Concentration / mM	Deviation / %
23	1	2934.22	2917.83	2926.02	21.95	4.59
	2	3212.70	3206.76	3209.73	24.07	4.67
	3	3134.85	3114.56	3124.70	23.44	1.89
	4	3188.45	3188.83	3188.64	23.91	3.98
	5	2942.53	2886.72	2914.62	21.86	4.96
11.5	1	1579.69	1537.92	1558.80	11.69	1.66
	2	1531.51	1527.48	1529.50	11.47	0.25
	3	1574.16	1583.83	1579.00	11.84	2.98
	4	1512.7	1461.79	1487.24	11.15	3.01
	5	1543.64	1560.67	1552.16	11.64	1.23
5.75	1	805.08	804.13	804.60	6.03	4.95
	2	760.94	754.67	757.80	5.68	1.16
	3	740.56	749.39	744.98	5.59	2.83
	4	775.47	783.16	779.32	5.84	1.65
	5	782.41	783.81	783.11	5.87	2.14
2.875	1	393.61	403.88	398.74	2.99	4.02
	2	394.04	381.97	388.00	2.91	1.22
	3	396.86	398.83	397.84	2.98	3.79
	4	373.23	372.17	372.70	2.80	2.77
	5	394.97	390.65	392.81	2.95	2.47
1.15	1	158.84	160.25	159.54	1.20	4.05
	2	152.13	165.27	158.70	1.19	3.50
	3	159.54	151.25	155.40	1.17	1.34
	4	157.57	145.79	151.68	1.14	1.08
	5	153.84	155.62	154.73	1.16	0.91

5. Guest transport kinetic studies

5.1. Kinetics experimental setup

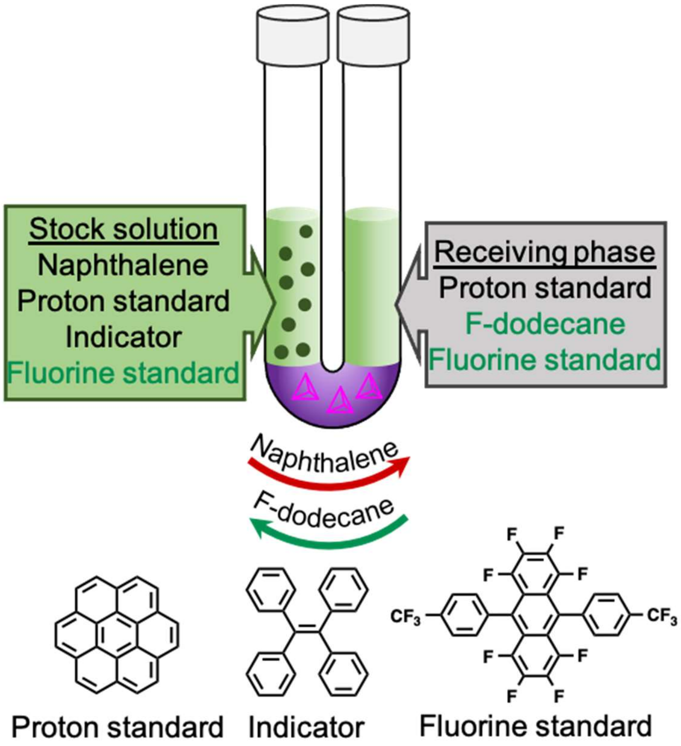

Figure S17: Experimental scheme to monitor the transport of naphthalene and 1-fluorododecane in the triphasic system

An aqueous solution of either cage **1** or cage **2** (2 mM, 2.5 mL) was employed as a membrane layer, separating a feedstock arm and a receiving arm. The membrane must be immiscible with the stock and receiving organic layers, and impermeable to the guest compounds. To satisfy these conditions, water was chosen as the medium for the liquid membranes. Because cage **1** and **2** must be soluble in water, the cages were prepared with SO_4^{2-} counterions. The receiving arm contained a neat dodecane solution with coronene (0.25 mM) standard. The feedstock arm contained a naphthalene solution (10 mM, 2 mL) in dodecane. Knowing for unbinding to the cages and insoluble in water, coronene (0.25 mM) and tetraphenylbenzene (0.5 mM) were introduced as the ^{1}H NMR concentration standard and an indicator for the experiment. As stirring at the bottom of the tube could cause the disturbance of the layers and undesirable

mixing of the solutions in two arms, any deviation in the known concentration of tetraphenylbenzene (0.5 mM) could signal a failed experiment. If the solution from the feedstock was accidentally mixed into the receiving arm, tetraphenylbenzene ^{1}H NMR signal will show in the receiving arm. If the solution from the receiving arm was accidentally mixed into the feedstock arm, the tetraphenylbenzene concentration in the feedstock arm was diluted to less than 0.5 mM. The experiment was stirring with a magnetic bar at a constant speed of 250 rpm, which was observed to avoid disturbance to the solutions in the two arms.

As dodecane has been reported to bind to cage **1** in fast exchange[1], this solvent could potentially compete with and displace encapsulated naphthalene. To investigate if dodecane was transported by the cages in the triphasic system, 1-fluorododecane (10 mM) was introduced to the receiving arm. The transport of 1-fluorododecane from the receiving to the feedstock arm would be quantified by referencing to octafluoro-9,10-bis[4-(trifluoromethyl)phenyl]-anthracene, a ^{19}F NMR concentration standard, present in both arms. The 1-fluorododecane concentration in the two arms were monitored by ^{19}F NMR.

While naphthalene was transported from the feedstock to the receiving arm, no transport of 1-fluorododecane was observed in the reverse direction, indicating that neither cage **1** nor **2** is appropriate carriers for dodecane (Figure S19, S21). This result suggests that dodecane does not act as a competing guest within our systems.

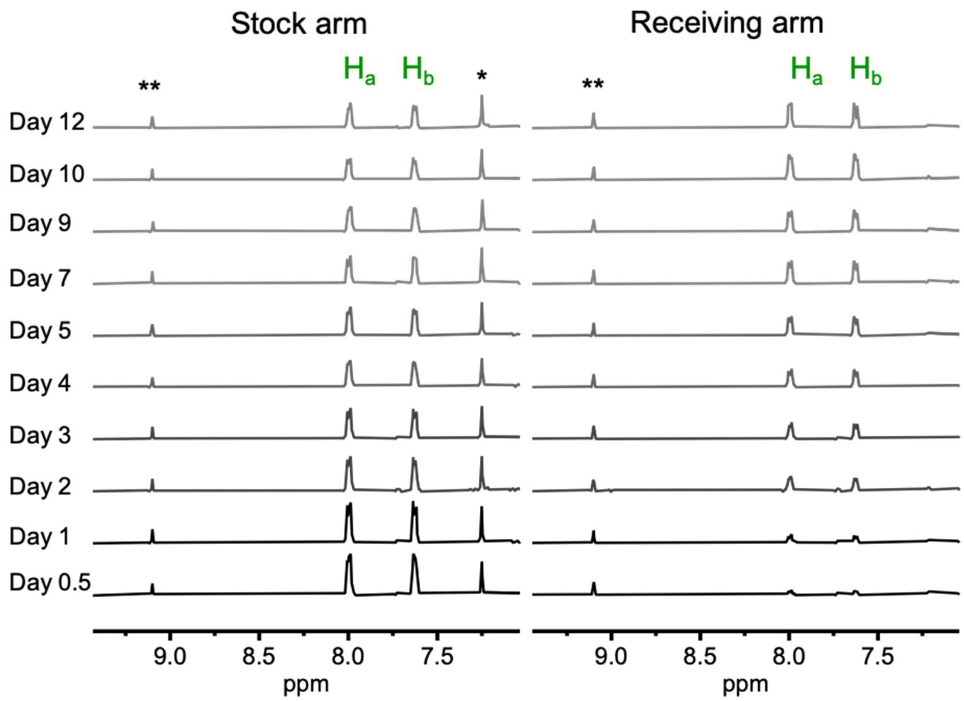

Figure S18: ^{1}H NMR spectra (500 MHz, D_2O, 298 K) of the feedstock phase and the receiving arms reflecting the change in naphthalene concentration (H_a and H_b) over time when filtered by cage **1** (** = coronene; * = tetraphenylethylene).

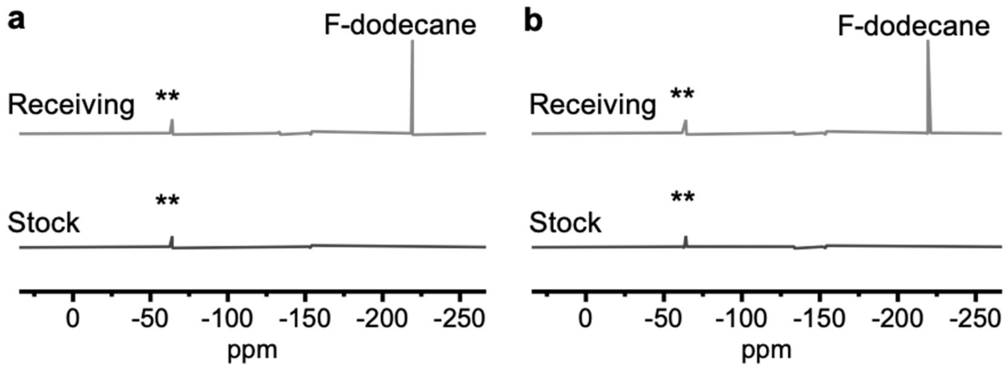

Figure S19: ^{19}F NMR spectra (400 MHz, D_2O, 298 K) of the feedstock and the receiving arms filtered by cage **1**, **a**, before and **b**, after the kinetic experiment. No 1-fluorododecane was transported. (** = octafluoro-9,10-bis[4-(trifluoromethyl)phenylanthracene).

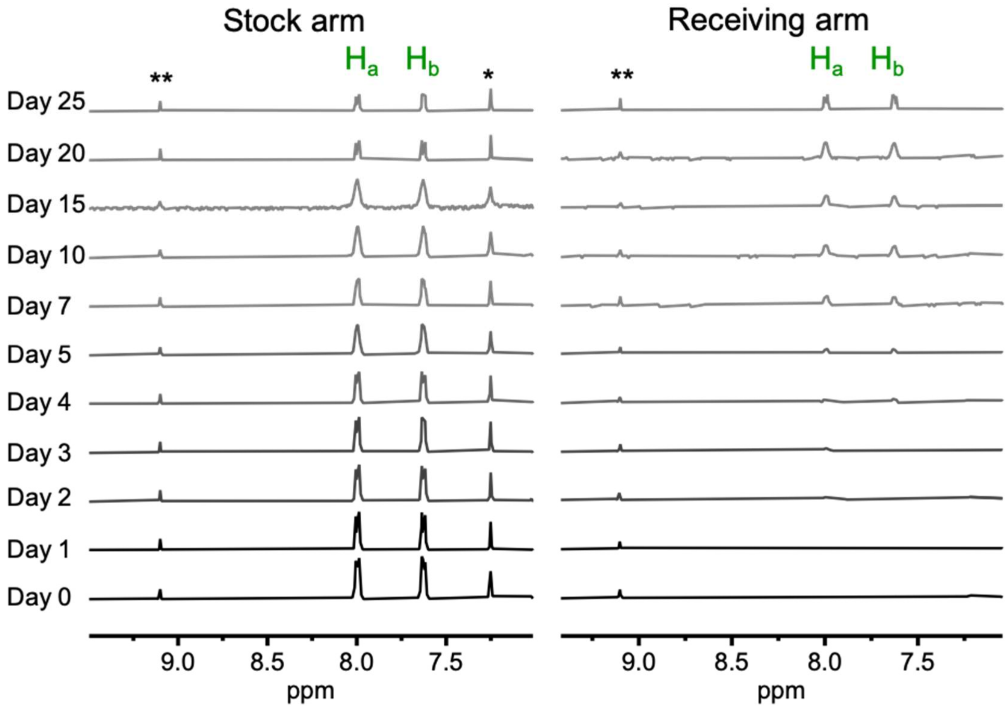

Figure S20: ^{1}H NMR spectra (500 MHz, D_2O, 298 K) of the feedstock phase and the receiving arms reflecting the change in naphthalene concentration (H_a and H_b) over time when filtered by cage **2** (** = coronene; * = tetraphenylethylene).

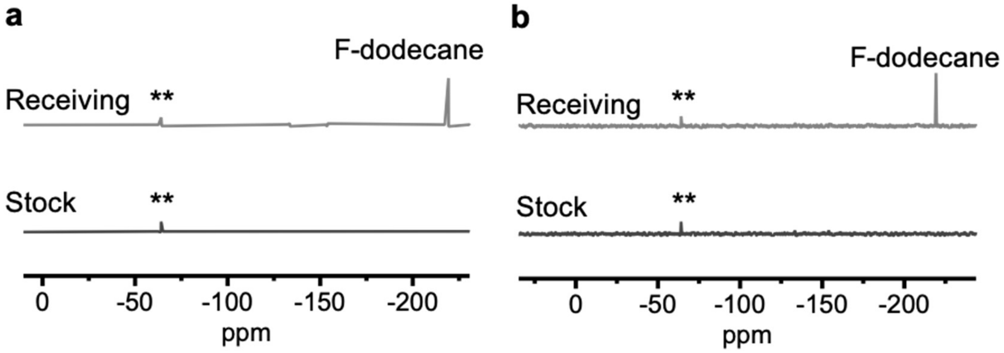

Figure S21: ^{19}F NMR spectra (400 MHz, D_2O, 298 K) of the feedstock and the receiving arms filtered by cage **2**, **a**, before and **b**, after the kinetic experiment. No 1-fluorododecane was transported. (** = octafluoro-9,10-bis[4-(trifluoromethyl)phenylanthracene).

5.2. Modelling the transport mechanism

The transport data were modeled with a three state model in which the naphthalene is distributed between the feedstock, receiving arm, and in the aqueous cage layer. This transport model can be expressed as an equilibrium between three states:

$$N_A \underset{T_r}{\overset{T_f}{\rightleftarrows}} N_B \underset{T_f}{\overset{T_r}{\rightleftarrows}} N_C \tag{9}$$

where N_A is the concentration of naphthalene in the feedstock arm, N_B is the concentration of naphthalene in the cage (in aqueous solution), and N_C is the concentration of naphthalene in the receiving arm. By the principle of microscopic reversibility, the transfer of naphthalene from the feedstock to the cage layer ($N_A \rightarrow N_B$) is identical to the transfer from the receiving to cage layer ($N_C \rightarrow N_B$), thus their rate constants are identical. Likewise, the rate constant for $N_B \rightarrow N_A$ is identical that for $N_B \rightarrow N_C$. This transport mechanism does not specify any particular set of bond-making or bond-breaking mechanism for ingress and egress of guest, but rather focuses on macroscopic movement of material across an aqueous membrane. One assumption of this transport model is that the rate of diffusion of encapsulated naphthalene through the water is much faster than either crossing the phase boundary or the encapsulation process itself.

The rate of change of the concentration of these three species are governed by the following three differential equations.

$$\frac{dN_A}{dt} = -k_f N_A(t) + k_r N_B(t) \tag{10}$$

$$\frac{dN_B}{dt} = +k_f N_A(t) - 2k_r N_B(t) + k_f N_C(t) \tag{11}$$

$$\frac{dN_C}{dt} = +k_r N_B(t) - k_f N_C(t) \tag{12}$$

Using the appropriate boundary conditions ($N_A = N_{A0}$, $N_B = 0$, $N_C = 0$, *i.e.,* naphthalene is in only the feedstock phase at time = 0), leads to the following analytical solutions:

$$N_A = \frac{\left(k_f\left(1+e^{-2k_r t}\right)+2k_r\left(1+e^{k_f t}\right)\right)e^{-k_f t}}{2\left(k_f+2k_r\right)}N_{A0} \tag{13}$$

$$N_B = \frac{k_f\left(1-e^{-\left(k_f+2k_r\right)t}\right)}{k_f+2k_r}N_{A0} \tag{14}$$

$$N_C = \frac{\left(-k_f\left(1-e^{-2k_r t}\right)-2k_r\left(1-e^{k_f t}\right)\right)e^{-k_f t}}{2\left(k_f+2k_r\right)}N_{A0} \tag{15}$$

The concentration of cage-encapsulated naphthalene, N_B, was not independently measured by NMR, but rather determined from mass balance: $N_B = (V_{stock}/V_{cage}) \times (N_{A0} - N_A - N_C)$. The factor V_{stock}/V_{cage} (= 2.0 mL/2.5 mL = 0.8 in the present study) arises because the aqueous and organic phases have different volumes resulting in a concentration gradient that contributes to the thermodynamic driving force for migration between the organic to aqueous layers.

The concentration-time data for N_A and N_C were fit simultaneously using a nonlinear least-squares fit as implemented on *Mathematica.*

```
Remove["Global`*"];

(* Read in data from file in csv format.  Format - column 1: time, col 2: [NA], col 3: σ[NA],
*)
(* col 4: [NC], col 5: σ[NC], col 6: [NAC], col 7: σ[NAC], col 8: [Nb], col 9: [Nb],          *)
glycerol = Import["/path/filename.csv", "Data"];
l = Length[filename];
NAdat = Table[{glycerol[[i,1]],glycerol[[i,2]]},{i,2,l}];
NCdat = Table[{glycerol[[i,1]],glycerol[[i,4]]},{i,2,l}];
ACdat = Table[{glycerol[[i,1]],glycerol[[i,6]]},{i,2,l}];
Nbdat = Table[{glycerol[[i,1]],glycerol[[i,8]]},{i,2,l}];

(* Initialize variables and define functions *)
NA0 = 10.2;
NA[t_]:=(E^(-kf*t)(kf*(1+E^(-2*kr*t))+2*kr(1+E^(kf*t)))*NA0)/(2*(kf+2*kr));
NC[t_]:=(E^(-kf*t)(kf*(E^(-2*kr*t)-1)+2*kr(E^(kf*t)-1))*NA0)/(2*(kf+2*kr));
NB[t_]:=(0.8(1-E^(-(kf+2*kr)t))*kf*NA0)/(kf+2*kr);
Ntot[t_]:= NA[t]+NB[t]+Nc[t];
```

```
(* Combine data sets and define fitting function *)
NACdat = Join[{1,Sequence@@#}&/@NAdat,{2,Sequence@@#}&/@NCdat];
modelsum[index_,t_]:=(KroneckerDelta[index-1]*NA[t])+(KroneckerDelta[index-2]*NC[t]);

(* Nonlinear fit which vary parameters kf and kr so as to minimize deviations *)
fitNAC = NonlinearModelFit[NABdat,modelsum[index,t],{{kf,0.4},{kr,1.4}},{index,t}]
fitNAC["RSquared"]
fitNAC["ParameterTable"]
MatrixForm[fitNAC["CorrelationMatrix"]]
```

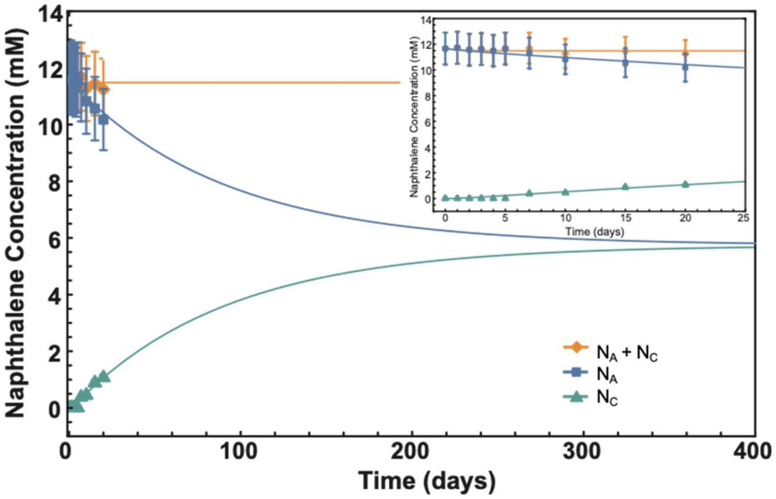

Figure S22: Control experiment of naphthalene transporting from the feedstock to the receiving arm. Instead of the cage solutions, deionized water (2.5 mL) was used as to separating the two arms. The data were fitted with the equations used for cage **1** and **2** experiments, suggesting that it may require more than 400 days for the naphthalene concentration in both arms to reach an equilibrium value through diffusion.

The rate constants for the transport of naphthalene across the aqueous barrier via cages **1** and **2** are given in Table S2. The *Mathematica* procedure yields a first order rate constant for the transfer of naphthalene from dodecane into cage (k_f^{obs}) and the reverse reaction from cage back into dodecane (k_f^{obs}). Dividing the observed rate constant k_f^{obs} by the U-tubes' cross-sectional area ($\pi r^2 = 1.13$ cm^2) and by the cage concentration (2.0 mM) gives a transport constant T_f with a unit of mM^{-1}·day^{-1}·cm^{-2} for transport of naphthalene to the cage according to the following rate law:

$$J_f = T_f[N][cage] \quad (16)$$

where J is the molar flux and N is naphthalene concentration in either the feedstock or receiving arm. Similarly, dividing the observed rate constant k_f^{obs} by the cross-sectional area gives a transport constant T_r a unit of day^{-1}·cm^{-2} for the transport of naphthalene back into organic solvent according to the following rate law:

$$J_r = T_r[N_B] \tag{17}$$

Table S2. Naphthalene transport parameters through the aqueous layers by cage **1** and **2**.

	k_f^{obs} (day^{-1})	k_f^{obs} (day^{-1})	T_f (mM^{-1}·day^{-1}·cm^{-2})	T_r (day^{-1}·cm^{-2})	R^2
Cage **1**	0.355 ± 0.007	14 ± 6	0.157 ± 0.003	12 ± 5	0.9994
Cage **2**	0.101 ± 0.003	0.16 ± 0.01	0.045 ± 0.001	0.14 ± 0.01	0.9996
Control	0.0111± 0.0008	0.3 ± 0.2	0.0049 ± 0.0004	0.3 ± 0.2	0.9996

The ratio T_f/T_r (= K_{eq}) is a measure of the thermodynamic preference for naphthalene to be sequestered in the cage compared to being dissolved in dodecane. For cage **1**, K_{eq} = 0.013, whilst for cage **2**, K_{eq} = 0.32. Although the rate of transport is faster with the more open glycerol cage **1**, the K_{eq} suggests triazine cage **2** appears to be the better binder of naphthalene. In both cases however naphthalene prefers to be dissolved in dodecane rather than being encapsulated by the cage (K_{eq} < 1). Although guest uptake by the cage is not favourable compared to solvation in dodecane, this situation is advantageous because it ensures that naphthalene will be released into the receiving phase. Efficient release of cargo is one of the key design elements of this cage-assisted transport system. This analysis also indicates that although the transport mechanism is relatively slow, the flux can be improved by increasing cage concentration and the diameter of the U-tube.

To find 50 % depletion of naphthalene from the feedstock arm, we first calculate the total concentration drop from t = 0 to t = eq, $(N_A^0 - N_A^{eq})$, divide by 2, then subtract that fromN_A^0:

$$N_A^{50\%} = N_A^0 - \frac{(N_A^0 - N_A^{eq})}{2} = \frac{(N_A^0 + N_A^{eq})}{2} \tag{18}$$

Assuming $N_C^0 = 0$ at time = 0, the 50 % increase of naphthalene in the receiving arm is simply 1/2 (N_C^{eq}).

Table S3. 50% depletion of naphthalene from the feedstock arm and accumulation in the receiving arm

	N_A^0 (mM)	N_A^{eq} (mM)	½ (N_A^0 + N_A^{eq}) (mM)	$t_A^{50\%}$ (days)	N_C^{eq} (mM)	½ N_C^{eq} (mM)	$t_C^{50\%}$ (days)
Cage **1**	10.2	5.04	7.62	1.92	5.03	2.52	1.99
Cage **2**	11.1	4.2	7.66	5.17	4.2	2.11	9.41
Control	11.6	5.76	8.71	60.9	5.72	2.86	63.7

We envisaged that naphthalene transport by cage carriers across the liquid membrane occurs over four steps. (1) Naphthalene from the dodecane feedstock diffuses into the water layer, wherein (2) naphthalene becomes encapsulated within the hydrophobic cavity of the cage. (3) Encapsulated naphthalene is then transported across the liquid membrane. (4) Finally, naphthalene is released from the cage and extracted into the receiving phase.

6. Outlook on potential solvent choices for the receiving phase

Solvents that allow higher partition coefficients (P) for naphthalene and other guests would be beneficial for guest transport. As there would be a larger amount of guest released to the receiving arm, the guest filtering process would be more efficient. Currently, we use dodecane in both the stock and the receiving arms, so the best separation we can achieve is 50:50, when logP = 0. With different organic solvents in each arm, there can be a non-zero logP value across the organic solvents thus driving different final concentrations of guests in each arm.

Taking naphthalene as an example, a few hydrophobic solvents suggested below are good candidates for our system as they solubilize naphthalene and are immiscible with water. The partitioning coefficient P is defined as the concentration ratio of naphthalene in dodecane (stock arm) and the new solvents (receiving arm) at equilibrium. LogP values can be calcuated from the solvation energy (ΔG_S^0) for a given solute in two different solvents to give a $\Delta\Delta G_S^0$ as shown in Figure S23.

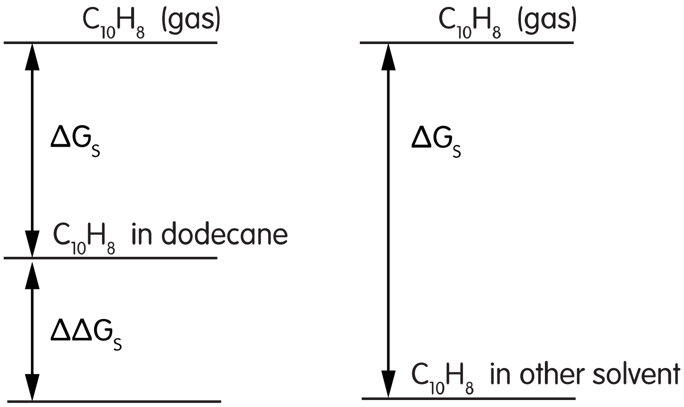

Figure S23: Calculation of logP using solvation energy for partitioning of naphthalene between dodecane and a second organic solvent.

The geometry of naphthalene was optimized using the B3LYP method and the 6-31G* basis set. Solvation energy was the calculated with the same method and basis set using the SM8 solvent model developed by Cramer and Truhlar.[7] LogP is then calculated using equation $logP = \left(\Delta G_S^{dodec} - \Delta G_S^{solv}\right)$ / RTln(10), and reported in Table S4.

Table S4. Calculated values P, log P and naphthalene concentration ratio in the two organic arms.

	Stock arm	Receiving arm	log P	P	Naphthalene concentration ratio (Stock/Receiving arm)
0	dodecane	dodecane	0	1	1/1
1	dodecane	decalin	–0.012	$10^{-0.012}$	1/ 0.97
2	dodecane	1-fluorooctane	+0.331	$10^{+0.331}$	2.14 / 1
3	dodecane	benzene	+0.659	$10^{+0.659}$	4.57 / 1
4	dodecane	diphenyl ether	+1.202	$10^{+1.202}$	15.91 / 1

With decalin in the receiving arm, P is almost equal to 1, so there will be no improvement compared to the dodecane/dodecane system. In the case of fluorooctane, we envisage that

naphthalene will favour arm B as its distribution ratio between fluorooctane and dodecane is 2:1. Naphthalene will thus be shifted preferentially to the receiving arm. Benzene would be a good solvent as it allows for the naphthalene distribution ratio between the two arms to increase to 4.6:1. However, benzene would be a guest for the cage. When present in excess, it would saturate the cage and hamper the transport of the target compounds through competition for binding.

Diphenyl ether will be a more suitable solvent as it has a larger size and allows for a considerable improvement in naphthalene distribution ratio between the two arms (16:1). As a result, using diphenyl ether in the receiving arm will provide a stronger driving force to transport more naphthalene across the cage membrane in a relatively faster process.

7. Naphthalene transported by different concentrations of cage 2

To investigate the effect of cage concentration on the rate of naphthalene transport, we monitored naphthalene building up in the receiving arm when varied concentrations of cage **2** were used in the membrane layer. The bottom of the tube was loaded with cage **2** solution at different concentrations of 0, 1, 2 and 4 mM. A dodecane solution (7 mL) containing naphthalene (10 mM), coronene (0.25 mM) and tetraphenylbenzene (0.5 mM) was introduced to the feedstock arm. Another solution of dodecane (7 mL) containing coronene (0.25 mM) was introduced as the receiving phase. The rate of naphthalene transported was monitored by ^{1}H NMR.

When higher concentrations of **2** were used, naphthalene was observed to transport more rapidly (Figure S24a), reflecting a shorter induction time required for naphthalene to be detected in the receiving arm. While the induction period lasted 3 days with 4 mM of **2**, decreasing the cage concentration to 0.5 mM caused the period to last up to 14 days. This result suggested that

introducing higher concentrations of cage generates more carriers within the liquid membrane and would allow higher rates of material transport.

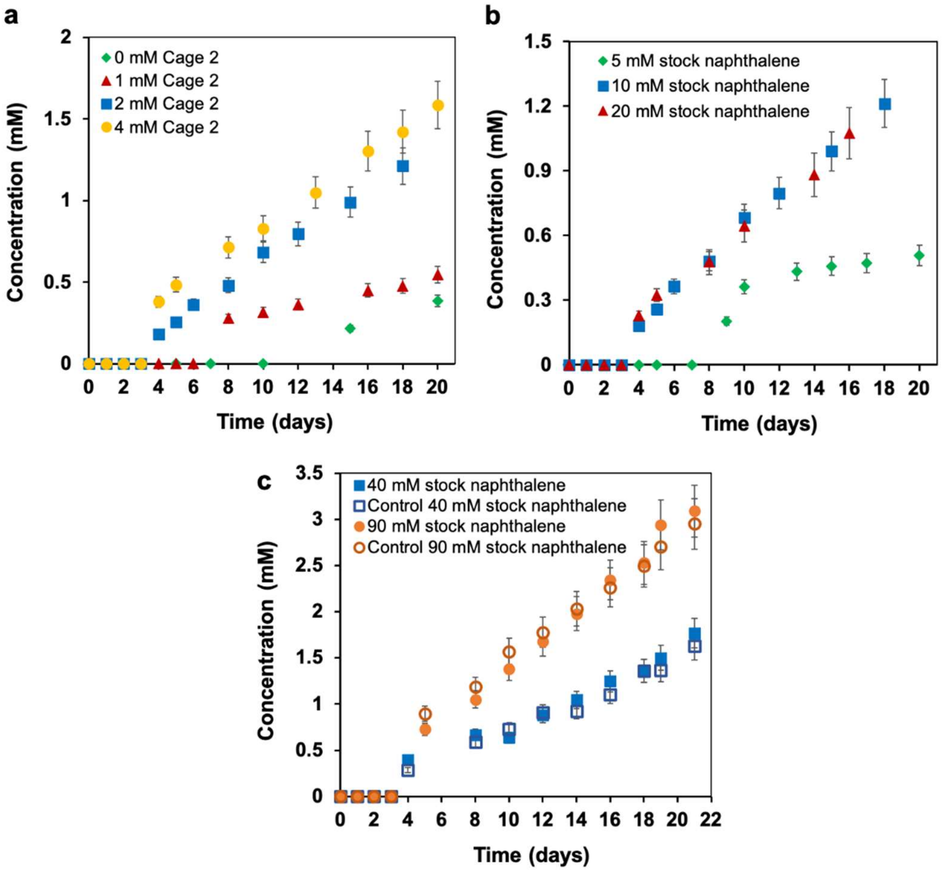

Figure S24: a, Naphthalene concentration accumulating in the receiving phases observed by ^{1}H NMR as functions of time when the concentration of cage **2** was varied. Faster transport of naphthalene was observed at higher concentrations of the cage. **b**, Naphthalene concentration accumulating in the receiving arm observed by ^{1}H NMR when varied concentration of feedstock naphthalene was introduced. A significant improvement in naphthalene transport rate was observed when the feedstock concentration was doubled from 5 to 10 mM. However, no significant increase in naphthalene transport rate was achieved when the feedstock increased to 20 mM. **c**, Further increasing the feedstock naphthalene concentration to 40 and 90 mM do not accelerate transport.

8. Naphthalene transported at varied feedstock concentration

Similarly, we investigated the effect of feedstock concentration on the rate of naphthalene transport. Naphthalene solution of 5, 10, 20, 40 and 90 mM was introduced to the feedstock arm as feedstock phases (7 mL). Coronene (0.25 mM) and tetraphenylbenzene (0.5 mM) were

used as an ^1H NMR standard and an indicator. The membrane layer was prepared with cage **2** (2 mM, 2.5 mL). A layer of dodecane (7 mL) containing coronene (0.25 mM) was added to the receiving arm. Naphthalene transported was monitored by ^1H NMR.

We expected that passive diffusion of naphthalene through the aqueous layers would increase when higher feedstock naphthalene concentration is used. To control for this effect, we ran parallel experiments in the absence of the cage carriers using deionized water. To better compare the rates of naphthalene transported by **2**, we subtracted the rates of passive transport obtained in our control experiments from the rates of transport obtained in the corresponding one with cage **2** (Figure S24b). With higher naphthalene concentrations, there were more cargoes available to be taken up by **2**, thus resulting in more rapid transport. When the feedstock naphthalene concentration was increased from 5 mM to 10 mM, large gains in the naphthalene concentration transported to the receiving arm were achieved. Increasing naphthalene concentration to 20 mM, however, did not significantly improve the active transport. These results suggested that most of the active **2** carriers were fully engaged in naphthalene transport process when the feedstock naphthalene concentration is above 10 mM.

When naphthalene concentration increased to 40 and 90 mM, the amount transported by **2** did not show any further increase compared to the corresponding control experiments (Figure S24c). These results suggested that when the feedstock guest concentration was very high, diffusion action driven by the chemical concentration gradient between the arms outdid transport by cage **2**.

9. Experimental setup for selective guest filtering experiments

Stage 1 filtration took place with cage **1** (2.0 mM, 2.5 mL) solution. As dodecane is a spectator within our systems, we continued to use this solvent for both the feedstock and receiving solutions. A feedstock solution containing naphthalene, mesitylene, *cis*-stilbene and tri-

isopropyl benzene (30 mM each, 2 mL) in dodecane was added to the feedstock arm. Tetraphenylbenzene was added as an indicator for the experiment. Dodecane (2 mL) was used as the receiving phase. The transport of the four guests was monitored in both arms by ^{1}H NMR, referencing to coronene (0.25 mM) (Figure S25).

The four guest compounds were chosen based on their sizes. In terms of thermodynamic binding, *cis*-stilbene binds strongly whereas triisopropylbenzene binds weakly to cage **1**[1] (ESI, Section 10.2)

To investigate the guest diffusion effect, a control experiment was set up with DI water used instead of **1**. The experimental setup was the same as the actual experiment. After an identical experiment duration of 43 days, only 15 % of naphthalene and 4.4 % of mesitylene were observed to transfer to the feedstock arm by diffusion action. Nevertheless, *cis*-stilbene and triisopropylbenzene were not observed to diffuse in the system.

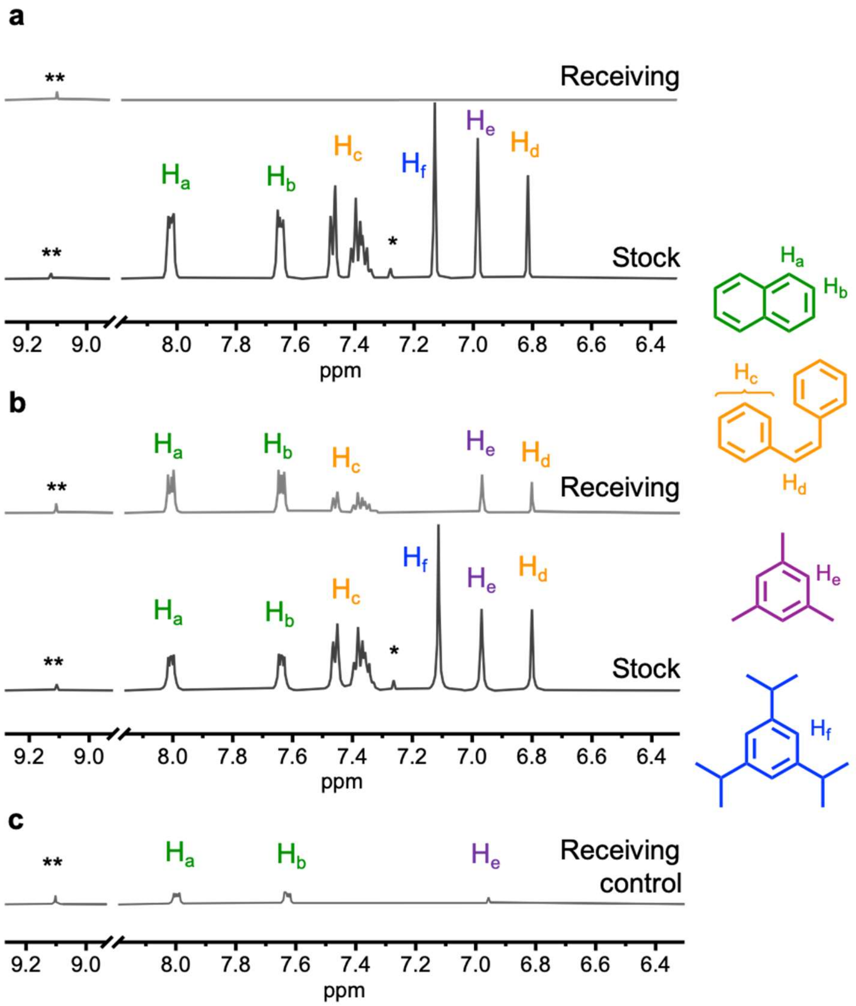

Figure S25: ^{1}H NMR spectra (500 MHz, D_2O, 298 K) of the guest transport in stage 1 by cage 1. **a**, Measurement at day 0 with all the guests occupying the feedstock arm. No guest signal was detected in the receiving arm. **b**, Measurement in both arms after 43 days. Naphthalene, mesitylene and *cis*-stilbene was transported while triisopropylbenzene was not. **c**, Naphthalene and mesitylene were observed to diffuse to the receiving phase in the control experiment in the absence of **1**. (** = coronene at 9.1 ppm; * = tetraphenylethylene).

Stage 2 filtration was caried out with cage **2** (2.0 mM, 2.5 mL) solution. In a separate U-tube, the solution (1 mL) extracted from the receiving arm in stage 1 was added as a new feedstock phase. The layer contained naphthalene (15 mM), mesitylene (6.3 mM) and *cis*-stilbene (4.7 mM). Tetraphenylethylene (0.5 mM) was added as an indicator. A new dodecane solution (1

mL) containing coronene (0.25 mM) was added as a new receiving phase to collect guests transported by cage **2**.

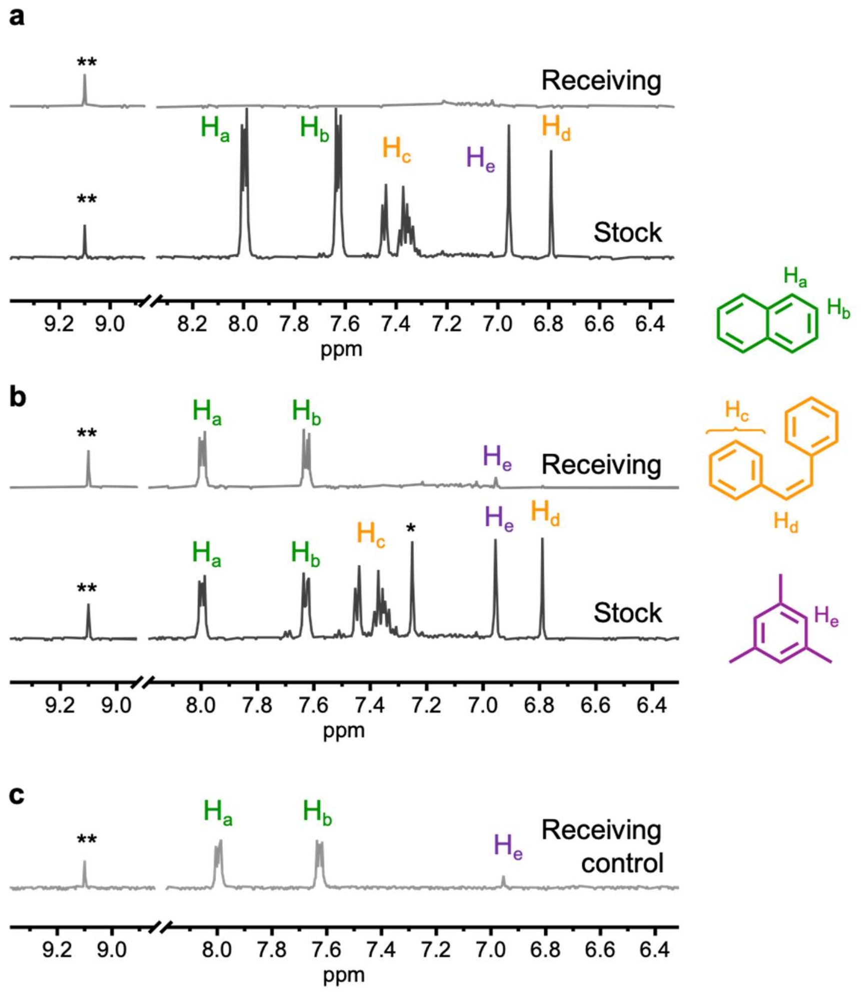

Figure S26: ^{1}H NMR spectra (500 MHz, D_2O, 298 K) of guest transport by cage 2 in stage 2. a, Measurement at day 0 with naphthalene, mesitylene and *cis*-stilbene present in the new feedstock arm. No guest was detected in the receiving arm. **b**, Except *cis*-stilbene, naphthalene and a negligible amount of mesitylene was observed to transport to the receiving arm after 25 days. **c**, A limited amount of naphthalene and mesitylene were observed to diffuse to the receiving arm in the control experiment without **2**. (** = coronene; * = tetraphenylethylene)

A control experiment investigating the diffusion of the guest in stage 2 was carried out in the absence of **2**. A feedstock layer solution was prepared with dodecane containing naphthalene (15 mM), mesitylene (6.3 mM), *cis*-stilbene (4.7 mM) and tetraphenylethylene (0.5 mM). A layer of DI water was used as the membrane in place of cage **2**. Another dodecane solution was

added as a receiving phase. The guest transport was referenced to coronene (0.25 mM). After 25 days, a minute amount of naphthalene (1.3 mM) and mesitylene (0.2 mM) were observed to diffuse to the receiving phase.

Table S5. Guests filtered by the aqueous membranes of cage **1** and **2** in stages 1 and 2. The concentrations and percentages of the guests in the arms were identified after 43 days for stage 1 and 25 days for stage 2.

Compounds	Concentration after stage 1 by cage **1** / mM (Percentage / %) after 43 days		Concentration after stage 2 by cage **2** / mM (Percentage / %) after 25 days	
	Stock	Receiving	Stock	Receiving
Naphthalene	15 (50)	15 (50)	5.6 (19)	5.2 (17)
Mesitylene	19 (63)	6.3 (21)	4.6 (15)	0.3 (1.0)
Cis-stilbene	20 (67)	4.7 (16)	4.6 (15)	0 (0)
Triisopropylbenzene	29 (97)	0 (0)	0 (0)	N/A

10. Host-guest studies

10.1. Naphthalene encapsulation by 1 in water

Naphthalene ⊂ 1: To a solution of cage **1** (2 mM, 0.4 mL), naphthalene (0.56 mg, 4.4 mmol, 5.5 equiv.) was added. The mixture was sonicated briefly and stirred for 1 hour. ^{1}H NMR and ^{1}H DOSY NMR spectra of the sample were measured to characterise the naphthalene ⊂ **1** host-guest complex. The naphthalene ⊂ **1** signals were observed at 6.3 and 6.2 ppm. The host signals were also observed to shift with the singlet at 5.5 ppm broadened into the baseline. Due to the low solubility of naphthalene in water, the unbound guest signals were not observed.

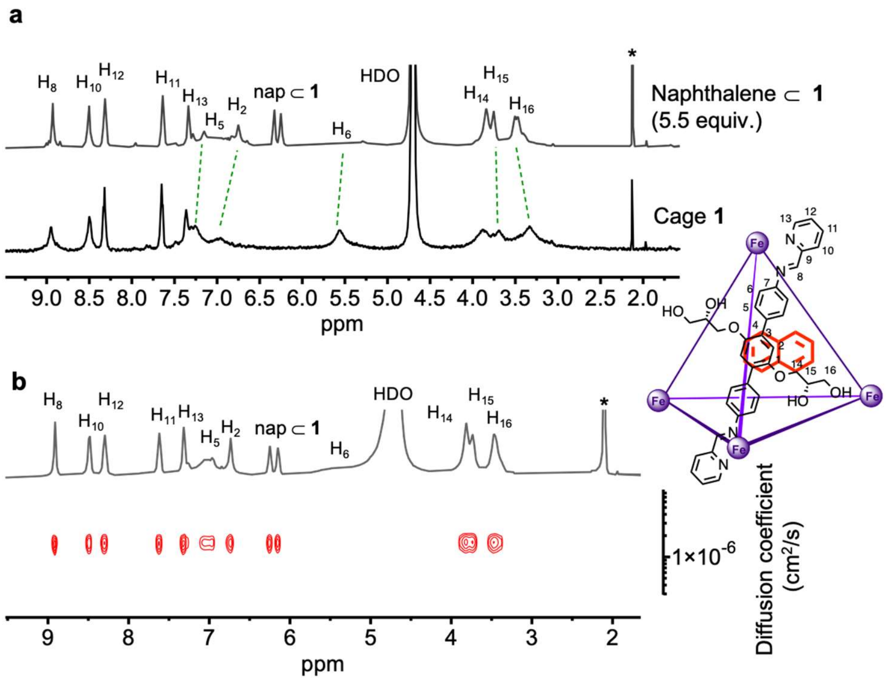

Figure S27: **a**, ^{1}H NMR (500 MHz, D_2O) spectra of cage **1** and the naphthalene ⊂ **1** host-guest complex. (* = CH_3CN reference signal). **b**, ^{1}H DOSY NMR (500 MHz, D_2O) spectrum of naphthalene ⊂ **1**, suggesting that the cage and encapsulated naphthalene diffused together as a single entity.

10.2. Hierarchy of guest binding to cage 1 in water

Due to the low solubilities of the aromatic guests in water, their binding constants to cage **1** could not be determined. In order to evaluate the relative binding affinities of the guests to cage **1**, we conducted an experiment to monitor the displacement of weak binding guests by stronger ones. To cage **1** solution in D_2O (2 mM, 1 mL), a sequence of guests, in the order of triisopropylbenzene (1.7 µL, 10 equiv.), *cis*-stilbene (2 µL, 11 equiv.), mesitylene (1 µL, 9.6 equiv.) and naphthalene (1.4 mg, 11 equiv.) were added. After the addition of each guest, the mixture was allowed to stir for 1h for the initial triisopropylbenzene, and then overnight for other guests to ensure guest exchange reached equilibrium. The sample was then centrifuged to separate the free organic guests from the aqueous cage solution. The ^{1}H NMR spectra of the samples were subsequently measured and compared to the reported spectra of triisopropylbenzene ⊂ **1** (ref. 1, ESI Section 8.2, pages S131-S132, Figure S205)[1], *cis*-stilbene

$\subset$ **1** (ref. 1, ESI Section 8.2, Page S150-S151, Figure S243)[1], mesitylene $\subset$ **1** (ref. 1, ESI Section 8.2, Page S115, Figure S172)[1] and naphthalene $\subset$ **1** (current study, ESI section 10.1, Page S37, Figure S27). Acetonitrile was added as a reference because this compound was not encapsulated by the cage and because its ^{1}H NMR spectrum measured in D_2O displays only a singlet peak at 2.15 ppm which does not overlap with any other guest signals. After each NMR measurement, the cage solution was recombined with the centrifuged layer containing the free guests, followed by the addition of a new guest at the beginning of a new guest replacement cycle.

Upon the addition of triisopropylbenzene, the ^{1}H NMR peak signals of **1** were observed to shift. Notably, the peak at 5.5 ppm was observed to broaden, signalling that guest binding had taken place. The peaks observed at 5.96 and 0.83 ppm was assigned to the encapsulated triisopropylbenzene. When *cis*-stilbene was added to the solution, the encapsulated triisopropylbenzene peak disappeared. Instead, bound *cis*-stilbene signals were observed at 6.7, 6.14 and 5.52 ppm, indicating that *cis*-stilbene has displaced triisopropylbenzene from **1**. Upon the addition of mesitylene, the encapsulated mesitylene peak was observed together with the encapsulated *cis*-stilbene signals, suggesting mesitylene and cis-stilbene had similar binding affinity cage to **1**. Naphthalene was observed to completely replace *cis*-stilbene and most of mesitylene when added in excess.

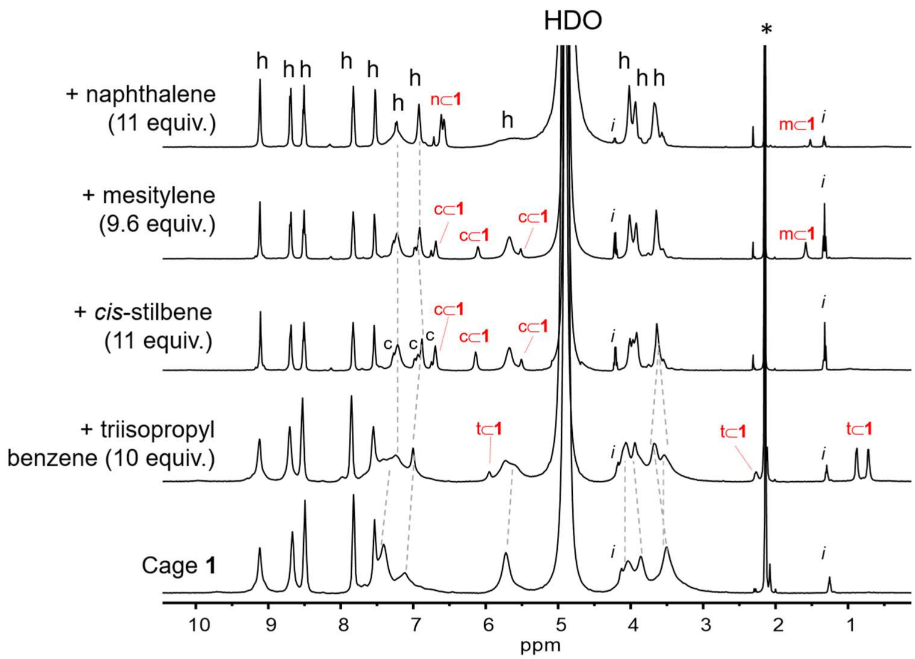

Figure S28: ^{1}H NMR monitoring relative binding strength of naphthalene, mesitylene, *cis*-stilbene and triisopropylbenzene to cage **1** in water. Naphthalene, mesitylene, *cis*-stilbene and triisopropylbenzene replaced one another when added progressively to the cage **1** solution in sequence. (h = host, c = free *cis*-stilbene and *i* = impurity, identified as ethyl acetate). Spectra were referenced CH_3CN (*)

10.3. Guest binding to cage 2 in water

Naphthalene ⊂ 2: To a solution of cage **2** (1 mM, 0.5 mL), naphthalene (0.25 mg, 2 mmol, 4 equiv.) was added. The solution was sonicated briefly and was allowed to stir for 1 h to ensure maximum encapsulation of the guest. The ^{1}H NMR spectrum of the sample was measured, referenced to ^{t}BuOH, to characterise the naphthalene ⊂ **2** host-guest complex. It was observed that naphthalene replaced the encapsulated 2-pyridinecarboxaldehyde templating the cage and was encapsulated in slow exchange on the NMR time scale. Further addition of naphthalene (10 equiv.) caused a complete displacement of 2-pyridinecarboxaldehyde from the cage and more prominent encapsulated naphthalene signals at –15.4 and –15.7 ppm were observed. No free naphthalene peak was observed due to its low solubility in water.

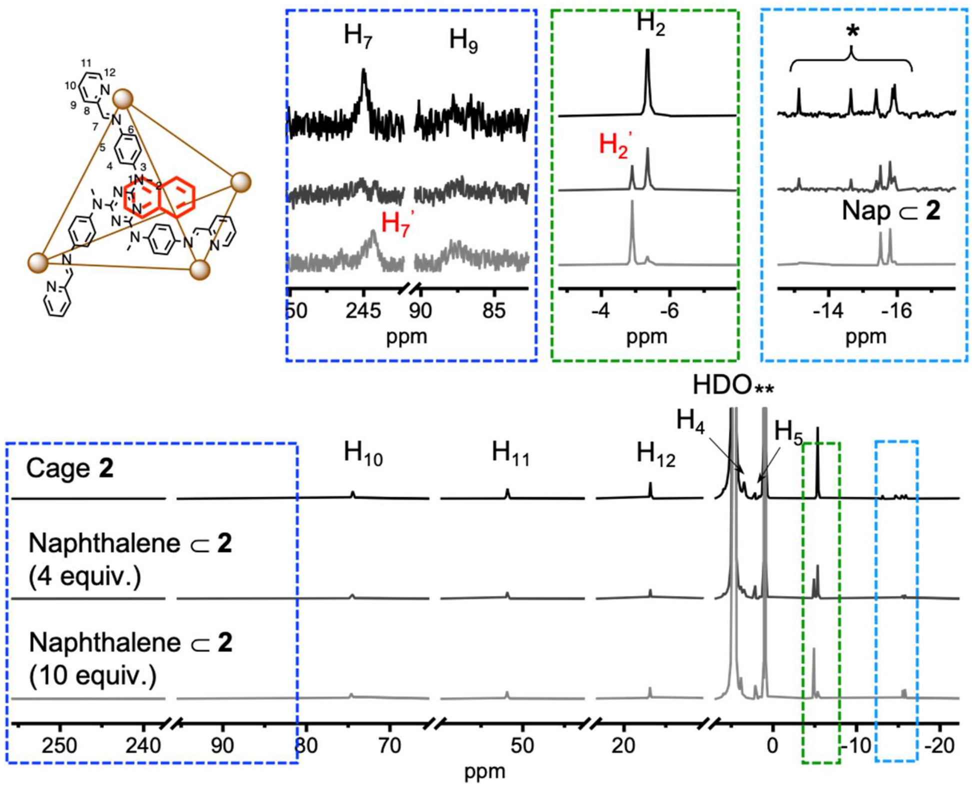

Figure S29: ^{1}H NMR (500 MHz, D_2O) spectra of cage **2** templated with 2-pyridinecarboxaldehyde and the naphthalene ⊂ **2** host-guest complex. (**) ^{t}BuOH reference signal, (*) encapsulated 2-pyridinecarboxaldehyde.

Mesitylene ⊂ 2: To a solution of cage **2** (1 mM, 0.5 mL), mesitylene (1.1 µL, 16 mM, 16 equiv.) was added. The solution was sonicated briefly and was allowed to stir for 1 h. The ^{1}NMR spectrum of the sample was measured, referencing to ^{t}BuOH, to characterise the mesitylene ⊂ **2** host-guest complex. The encapsulated mesitylene replaced the encapsulated 2-pyridinecarboxaldehyde template signals completely, suggesting complete encapsulation of mesitylene in the cage. Both the free and encapsulated mesitylene signals were observed indicating that mesitylene was encapsulated in slow exchange.

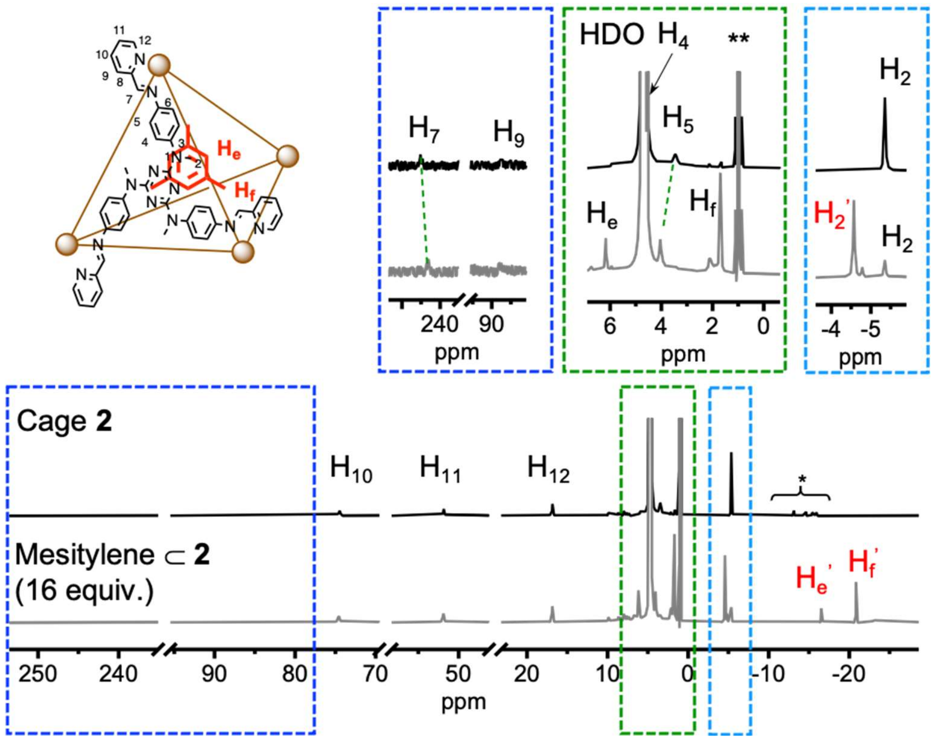

Figure S30: ^{1}H NMR (500 MHz, D_2O) spectra of cage **2** templating with 2-pyridinecarboxaldehyde and the mesitylene ⊂ **2** host-guest complex. (**) ^{t}BuOH reference signal, (*) the encapsulated 2-pyridinecarboxaldehyde. The H_2', H_e' and H_f' (red color coding) are the corresponding signals of the mesitylene ⊂ **2** host-guest complex.

Addition of *cis*-stilbene to 2: To a solution of cage **2** (1 mM, 0.5 mL), *cis*-stilbene (1 mg, 10 mM, 10 equiv.) was added. The solution was equilibrated for 24 hours. The ^{1}H NMR spectrum of the sample was measured, referenced to ^{t}BuOH. No evidence of guest encapsulation was observed.

10.4 Hierarchy of guest binding to cage 2 in water

In order to evaluate the relative binding affinities of naphthalene, mesitylene and *cis*-stilbene to cage **2**, we conducted an experiment to monitor the competing guest replacement, similar to that carried out for **1**. To a cage **2** solution in D_2O (2 mM, 0.5 mL), *cis*-stilbene, mesitylene and naphthalene were added in sequence and equilibrated at room temperature for 1 hour. The sample was subsequently measured by ^{1}H NMR after each guest addition.

No guest encapsulation was observed when *cis*-stilbene was added to the solution. When mesitylene was added, the mesitylene ⊂ **2** signals were observed at –16.6 and –20.9 ppm indicating slow exchange encapsulation. Upon naphthalene addition, the mesitylene ⊂ **2** signals disappeared. Instead, naphthalene ⊂ **2** signals were observed at –15.6 and –15.9 ppm, suggesting that naphthalene has replaced mesitylene from cage **2**.

The excess triisopropylbenzene, *cis*-stilbene and mesitylene exist in liquid form at room temperature, and formed a separate organic phase when introduced to the aqueous cage solution. The excess naphthalene also dissolved in this organic phase when added to the host-guest mixture. Signals from both the immiscible organic and aqueous cage phases were observed by ^{1}H NMR, giving rise to apparent free guest signals despite their low solubility in the aqueous layer.

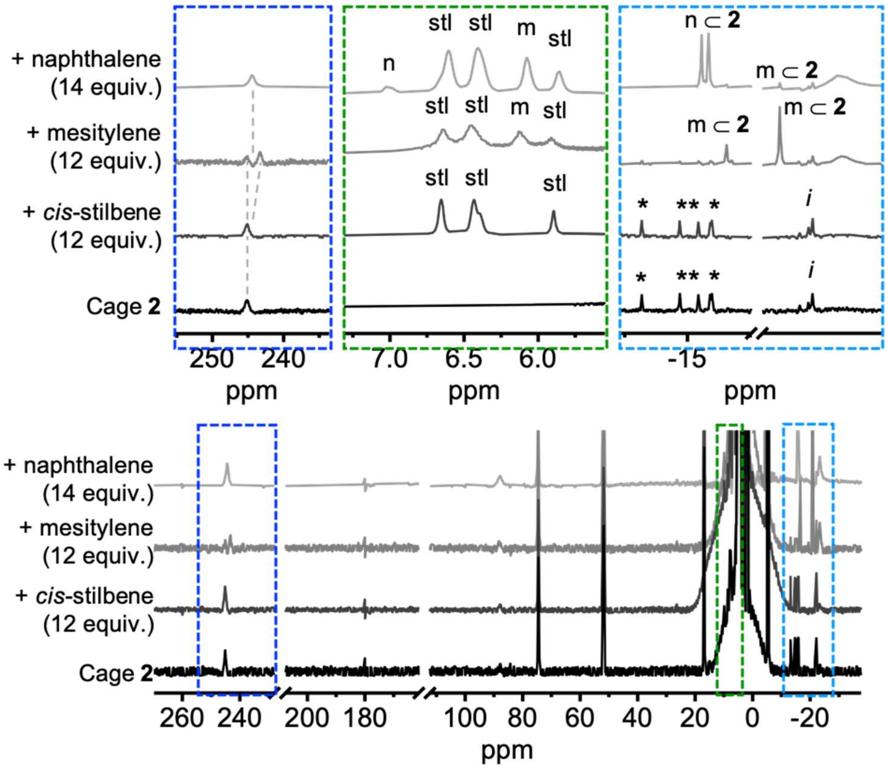

Figure S31: ^{1}H NMR (500 MHz, D_2O) competitive guest binding of naphthalene, mesitylene and *cis*-stilbene to cage 2 in water. *Cis*-stilbene does not show any sign of guest encapsulation to cage **2**. When mesitylene is added, the guest was observed to bind to cage **2** in slow exchange. The encapsulated mesitylene was subsequently replaced by naphthalene when

this guest was introduced to the mixture. The spectra were referenced to ^{t}BuOH (* = The encapsulated 2-pyridinecarboxaldehyde, *i* = unidentified minor impurity, n = free naphthalene, m = free mesitylene and stl = free *cis*-stilbene).

11. Independent transport of the four guests by cage 1

Four solutions of dodecane containing either naphthalene, mesitylene, *cis*-stilbene or tri-isopropylbenzene (30 mM each, 2mL) were introduced separately to identical U-tubes as the feedstock arms. Cage **1** solution (2 mM, 2.5 mL) were employed as the membrane layer. The guest transport to the receiving arms in the absence of the others was monitored by ^{1}H NMR for the first 7 days and after 43 days, same as the experiment time of stage 1 filtration.

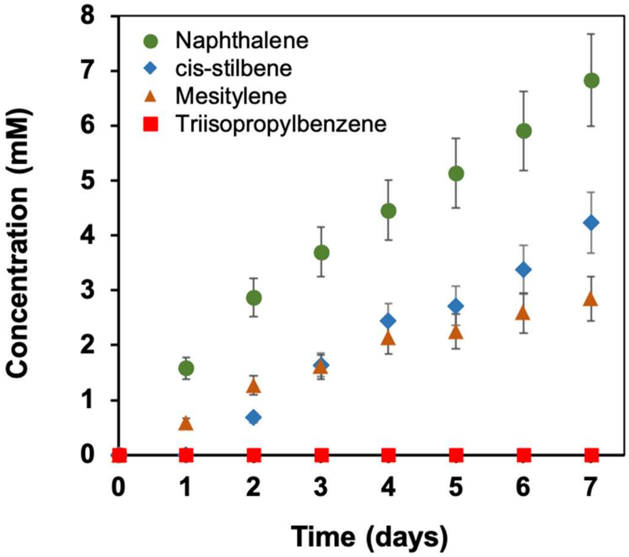

Figure S32: Plot showing independent transport of the four guests by cage **1** as a function of time, monitored by ^{1}H NMR.

Table S6. Concentration and percentage of guests transported by cage **1** in the absence of other competing guests in comparison to guest transported from the mixture in stage 1.

Compounds	Independent transported / mM (Percentage / %)		Transported in a mixture by / mM (Percentage / %)
	After 7 days	After 43 days	After 43 days
Naphthalene	6.4 (21)	15 (50) (After 33 days)	15 (50)
Mesitylene	2.8 (9.3)	7.1 (24)	6.3 (21)
Cis-stilbene	4.2 (14)	8.2 (27)	4.7 (16)
Tri-isopropylbenzene	0 (0)	0 (0)	0 (0)

12. X-ray crystallography

Cage **2**·[NTf_2^-]$_8$, an analogue of the sulfate salt **2**, was synthesised as follows. A mixture of **L2** (35.1 mg, 79 µmol, 4 equiv.), Co(NTf_2)$_2$.H_2O (52.1 mg, 82 µmol, 4.1 equiv.) and 2-pyridinecarboxaldehyde (23 µL, 242 µmol, 12.2 equiv.) was dissolved in CH_3CN (5 mL). The reaction was stirred at room temperature for 24 hours, yielding to a bright orange cage solution (4 mM). The cage was precipitated with Et_2O (35 mL), washed with further Et_2O (20 mL), and dried to air to yield **2**[NTf_2^-]$_8$ (84.5 mg, 16 µmol, 80 %). ^{1}H NMR (400 MHz, CD_3CN, 298 K) δ (ppm) 246.1 (H_7), 86.1 (H_9), 74.0 (H_{10}), 51.8 (H_{11}), 17.0 (H_{12}), 3.5 (H_4), −4.9 (H_5), −23.2 (H_2).

Crystals of **2**·[NTf_2^-]$_8$ in acetonitrile were grown by diffusion of diethyl ether into an acetonitrile solution of the complex. Data were collected at Beamline I19 of Diamond Light Source employing silicon double crystal monochromated synchrotron radiation (0.6889 Å) with ω and ψ scans at 100(2) K.[8] Data integration and reduction were undertaken with Xia2.[9,10] Subsequent computations were carried out using the WinGX-32 graphical user interface.[11] A multi-scan empirical absorption correction was applied to the data using the AIMLESS[12] tool in the CCP4 suite.[13,14] The structure was solved by intrinsic phasing using SHELXT[15] then refined and extended with SHELXL.[16] In general, non-hydrogen atoms with occupancies greater than 0.5 were refined anisotropically. Carbon-bound hydrogen atoms were included in idealised positions and refined using a riding model. Disorder was modelled using standard crystallographic methods including constraints, restraints and rigid bodies where necessary.

The crystals employed immediately lost solvent after removal from the mother liquor and rapid handling prior to flash cooling in liquid nitrogen was required to collect data. Despite these measures and the use of synchrotron radiation few reflections at greater than 1.0 Å resolution were observed and the data were trimmed accordingly. The tetrahedral assembly crystallised in the cubic space group I23 with 1/12 of the tetrahedron in the asymmetric unit.

Due to the less than ideal resolution and the significant thermal motion within the structure some bond length (DFIX) and angle (DANG) restraints were applied to the organic part of the structure and thermal parameter restraints (SIMU, RIGU) were applied to all atoms except for cobalt to facilitate anisotropic refinement.

The anions within the structure show evidence of substantial disorder. One triflimide lattice site was resolved in the asymmetric unit, located on a special position such that half the anion was present in the asymmetric unit. This anion was disordered over two separate locations and bond length and thermal parameter restraints were required to facilitate a reasonable refinement. The occupancies of the two disordered parts were allowed to freely refine. The thermal parameters of some atoms in the disordered triflimide anion remain higher than ideal, indicative of further minor disorder which could not be resolved.

No satisfactory model for the remaining anions (3.2 per Co_4L_4 assembly) could be found despite numerous attempts at modelling. Consequently, the SQUEEZE[17] function of PLATON[18] was employed to remove the contribution of the electron density associated with these remaining anions and further highly disordered solvent, which gave a potential solvent accessible void of 1316 Å^3 per unit cell (a total of approximately 561 electrons). The remaining anions are assigned as triflimide in the formula. The diffuse solvent molecules could not be assigned to acetonitrile or diethyl ether and are not included in the formula. Consequently, the molecular weight and density given above are underestimated.

The absolute configuration of the structure was confirmed using anomalous dispersion effects with the Flack parameter[19] refining to 0.04(3). We assume the bulk sample contains equal quantities of crystal of each enantiomer.

CheckCIF gives 1 level A alert and 5 B level alerts. These alerts all result from the limited resolution of the data (low resolution, low bond precision, low data to parameter ratio) and the

disordered triflimide anion (Large Hirshfeld Differences) as described above. Crystallographic data have been deposited with the CCDC (2047950).

Formula $C_{184}H_{144}Co_4F_{48}N_{56}O_{32}S_{16}$, *M* 5312.22, Cubic, space group *I*23 (#197), *a* 22.4786(2), *b* 22.4786(2), *c* 22.4786(2) Å, *V* 11358.2(3) Å^3, D_C 1.553 g cm^{-3}, *Z* 2, crystal size 0.10 by 0.05 by 0.05 mm, colour orange, habit prism, temperature 100(2) Kelvin, λ(synchrotron) 0.6889 Å, μ(synchrotron) 0.552 mm^{-1}, $T(\text{Analytical})_{min,max}$ 0.9859874635584035, 1.0, $2\theta_{max}$ 40.24, *hkl* range −22 22, −22 22, −21 21, *N* 18162, N_{ind} 1988 (R_{merge} 0.0602), N_{obs} 1164(I > 2σ(I)), N_{var} 267, residuals* $R1(F)$ 0.0851, $wR2(F^2)$ 0.2415, GoF(all) 0.929, $\Delta\rho_{min,max}$ −0.196, 0.397 e$^-$Å^{-3}.

*$R1 = \Sigma||F_o| - |F_o||/\Sigma|F_o|$ for $F_o > 2\sigma(F_o)$; $wR2 = (\Sigma w(F_o^2 - F_c^2)^2/\Sigma(wF_c^2)^2)^{1/2}$ all reflections

$w=1/[\sigma^2(F_o^2) + (0.1851P)^2]$ where $P = (F_o^2 + F_c^2)/3$

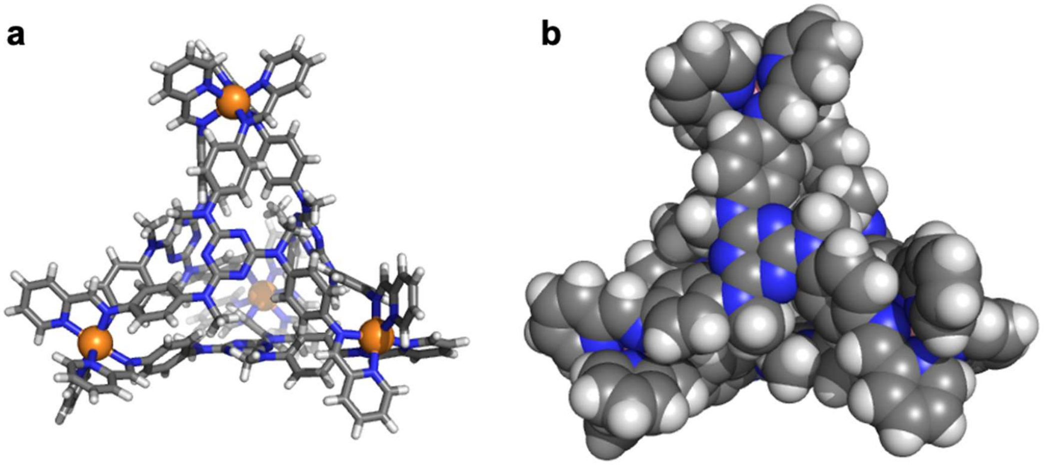

Figure S33: a, Stick and **b**, space-filling representation of cage **2** crystal structure. Counter ions and solvents are omitted for clarity.

13. References

(1) Bolliger, J. L.; Belenguer, A. M.; Nitschke, J. R. Enantiopure Water-Soluble Fe_4L_6 Cages: Host–Guest Chemistry and Catalytic Activity. *Angew. Chem. Int. Ed.* **2013**, *52* (31), 7958–7962, DOI: 10.1002/anie.201302136.

(2) Bolliger, J. L.; Ronson, T. K.; Ogawa, M.; Nitschke, J. R. Solvent Effects upon Guest Binding and Dynamics of a $Fe^{II}_4L_4$ Cage. *J. Am. Chem. Soc.* **2014**, *136* (41), 14545–

14553, DOI: 10.1021/ja5077102.

(3) McLeod, K.; Comisarow, M. B. Systematic Errors in the Discrete Integration of FT NMR Spectra. *J. Magn. Reson.* **1989**, *84* (3), 490–500, DOI: 10.1016/0022-2364(89)90115-7.

(4) Holzgrabe, U. Quantitative NMR Spectroscopy in Pharmaceutical Applications. *Prog. Nucl. Magn. Reson. Spectrosc.* **2010**, *57* (2), 229–240, DOI: 10.1016/j.pnmrs.2010.05.001.

(5) Simpson, A. J.; Brown, S. A. Purge NMR: Effective and Easy Solvent Suppression. *J. Magn. Reson.* **2005**, *175* (2), 340–346, DOI: 10.1016/j.jmr.2005.05.008.

(6) Howe, D. J.; Grommet, A. B.; Ho, J. B.; Perca, E. G.; Bolliger, J. L.; Nitschke, J. R. Anion Exchange Drives Reversible Phase Transfer of Coordination Cages and Their Cargoes. *J. Am. Chem. Soc.* **2018**, *140*, 14770–14776, DOI: 10.1021/jacs.8b07900.

(7) Cramer, C. J.; Truhlar, D. G. A. Universal Approach to Solvation Modeling. *Acc. Chem. Res*. **2008**, *41 (6),* 760-768. DOI: 10.1021/ar800019z.

(8) Allan, D. R.; Nowell, H.; Barnett, S. A.; Warren, M. R.; Wilcox, A.; Christensen, J.; Saunders, L. K.; Peach, A.; Hooper, M. T.; Zaja, L.; Patel, S.; Cahill, L.; Marshall, R.; Trimnell, S.; Foster, A. J.; Bates, T.; Lay, S.; Williams, M. A.; Hathaway, P. V; Winter, G.; Gerstel, M.; Wooley, R. W. A Novel Dual Air-Bearing Fixed-χ Diffractometer for Small-Molecule Single-Crystal X-Ray Diffraction on Beamline I19 at Diamond Light Source. *Crystals.* **2017**, 7, 336, DOI: 10.3390/cryst7110336.

(9) Winter, G.; Waterman, D. G.; Parkhurst, J. M.; Brewster, A. S.; Gildea, R. J.; Gerstel, M.; Fuentes-Montero, L.; Vollmar, M.; Michels-Clark, T.; Young, I. D.; Sauter, N. K.; Evans, G. DIALS: Implementation and Evaluation of a New Integration Package. *Acta Crystallogr. Sect. D Struct. Biol.* **2018**, *74* (2), 85–97, DOI: 10.1107/S2059798317017235.

(10) Winter, G. Xia2: An Expert System for Macromolecular Crystallography Data Reduction. *J. Appl. Crystallogr.* **2010**, *43* (1), 186–190, DOI: 10.1107/S0021889809045701.

(11) Farrugia, L. J. WinGX and ORTEP for Windows: An Update. *J. Appl. Crystallogr.* **2012**, *45* (4), 849–854, DOI: 10.1107/S0021889812029111.

(12) Evans, P. R.; Murshudov, G. N. How Good Are My Data and What Is the Resolution? *Acta Crystallogr. Sect. D Biol. Crystallogr.* **2013**, *69* (7), 1204–1214, DOI: 10.1107/S0907444913000061.

(13) Collaborative Computational Project, N. 4; IUCr. The CCP4 Suite: Programs for Protein Crystallography. *Acta Crystallogr. Sect. D Biol. Crystallogr.* **1994**, *50* (5), 760–763, DOI: 10.1107/S0907444994003112.

(14) Winn, M. D.; Ballard, C. C.; Cowtan, K. D.; Dodson, E. J.; Emsley, P.; Evans, P. R.; Keegan, R. M.; Krissinel, E. B.; Leslie, A. G. W.; McCoy, A.; McNicholas, S. J.; Murshudov, G. N.; Pannu, N. S.; Potterton, E. A.; Powell, H. R.; Read, R. J.; Vagin, A.; Wilson, K. S. Overview of the CCP4 Suite and Current Developments. *Acta Crystallographica Section D: Biological Crystallography*. International Union of Crystallography, **2011**, 67, 235–242, DOI: 10.1107/S0907444910045749.

(15) Sheldrick, G. M. SHELXT - Integrated Space-Group and Crystal-Structure Determination. *Acta Crystallogr. Sect. A Found. Crystallogr.* **2015**, *71* (1), 3–8, DOI: 10.1107/S2053273314026370.

(16) Sheldrick, G. M. Crystal Structure Refinement with SHELXL. *Acta Crystallogr. Sect. C Struct. Chem.* **2015**, *71* (1), 3–8, DOI: 10.1107/S2053229614024218.

(17) Van Der Sluis, P.; Spek, A. L. BYPASS: An Effective Method for the Refinement of Crystal Structures Containing Disordered Solvent Regions. *Acta Crystallogr. Sect. A* **1990**, *46* (3), 194–201, DOI: 10.1107/S0108767389011189.

(18) Spek, A. L. Single-Crystal Structure Validation with the Program PLATON. *J. Appl. Crystallogr.* **2003**, *36* (1), 7–13, DOI: 10.1107/S0021889802022112.

(19) Flack, H. D. Chiral and Achiral Crystal Structures. *Helv. Chim. Acta.* **2003**, *86* (4), 905–921, DOI: 10.1002/hlca.200390109.
